# Theoretical size distribution of fossil taxa: analysis of a null model

**DOI:** 10.1186/1742-4682-4-12

**Published:** 2007-03-22

**Authors:** William J Reed, Barry D Hughes

**Affiliations:** 1 Department of Mathematics and Statistics, University of Victoria, Victoria, British Columbia V8W 3P4, Canada; 2 Department of Mathematics and Statistics, University of Melbourne, Parkville, Victoria 3010, Australia

## Abstract

**Background:**

This article deals with the theoretical size distribution (of number of sub-taxa) of a fossil taxon arising from a simple null model of macroevolution.

**Model:**

New species arise through speciations occurring independently and at random at a fixed probability rate, while extinctions either occur independently and at random (background extinctions) or cataclysmically. In addition new genera are assumed to arise through speciations of a very radical nature, again assumed to occur independently and at random at a fixed probability rate.

**Conclusion:**

The size distributions of the pioneering genus (following a cataclysm) and of derived genera are determined. Also the distribution of the number of genera is considered along with a comparison of the probability of a monospecific genus with that of a monogeneric family.

## Background

Mathematical modelling of the evolution of lineages goes back at least to Yule[1] who developed the eponymous *Yule process *(homogeneous pure birth process) in which speciations occur independently and at random. Yule's model did not include extinctions *per se*, because he believed that they resulted only from cataclysmic events. This issue was discussed at greater length by Raup[2], who distinguished between background and episodic extinctions. Raup started from a homomogeneous birth-and-death process model (in which background extinctions occur, like speciations, independently and at random) for which he presented mathematical results, and described more complex models of extinction including episodic extinctions and a mixture of episodic and background extinctions. However he gave no mathematical results for these models. Stoyan[3] considered a time in-homogeneous birth-and death process, in which speciation and background extinction rates varied with time, based on the idea that younger paraclades have higher speciation rates, while older ones have higher background extinction rates.

There has been considerable discussion (*e.g*. Raup[2]; Patzkowsky[4]; Przeworski and Wall[5]) about the suitability of the null birth-and-death process model (with constant birth and death rates) as a macroevolutionary model of species diversification. In order to truly assess the validity of such a model it is necessary to have a full understanding of its properties which can then be compared with the fossil record. Specifically analysis is needed to generate hypotheses, which can be tested against available data. To date such an analysis is incomplete, relying on the partial analytic results of Raup[2] and the simulation results of Patzkowsky[4] and Przeworski and Wall[5].

Analytic results are clearly superior to simulation ones. In particular with analytic results for the size distribution of a clade one can fit the model via a multinomial likelihood, using observed size distributions, and thence test the adequacy of the underlying birth-and-death model using a statistical goodness-of-fit test. In addition analytic results are preferable to simulation ones, in that it is much easier to interpret a parametric formula than a collection of simulation results; and one does not have to distinguish between sampling variation due to a finite number of runs (noise) and signal.

It is the purpose of this paper to conduct a more thorough analysis of the birth-and-death model than that previosly carried out by Raup[2]. In particular we obtain results for size distributions of taxa and probabilities of monotypic taxa. In this paper we confine attention to obtaining analytic results and defer actual fitting and testing of the fit, using observed fossil data, to a future paper.

We develop the mathematical model presented by Raup[2] (and used in simulations by the above authors) to include the possibility of episodic, cataclysmic extinctions in which complete lineages are destroyed. We consider a hiearchy of models, which can include both cataclysmic and background extinctions of species and examine the resulting size distributions of extinct genera. We start (following section), as did Yule, by considering cataclysmic extinction only. Furthermore like Patzkowsky[4] and Przeworski and Wall [5], we assume that at any time an existing species can split, yielding a new species so radically different from existing ones that it becomes the founding member of a new genus. Thus we assume that the probability of a new genus being formed in an infinitesimal interval (*t*, *t *+ *dt*) is proportional to the total number of species in existence at time *t*. We derive results for the size distribution of extinct genera.

In the third and fourth sections we do the same assuming only background extinctions (but no cataclysmic extinction); and both cataclysmic and background extinctions (although the results here are limited). The fifth section is devoted to the distribution of the number of genera derived from the pioneering species and in the final section the probability of a monotypic genus is compared with that of a monogeneric family.

## Cataclysmic extinctions only

Yule[1] considered the evolution of a genus begining with one species at time *t *= 0, which thenceforth evolves as a homogeneous pure birth process (Yule process) with speciation rate (birth parameter) *λ*. He then showed that *N*_*t*_, the number of species alive at time *t*, follows a geometric distribution with probability mass function (pmf)

*p*_*n*_(*t*; 1) = Pr{*N*_*t *_= *n*|*N*_0 _= 1} = *e*^-*λt*^(1 - *e*^-*λt*^)^*n *- 1 ^    (1)

for *n *= 1,2,.... If instead there are initially *n*_0 _species then from standard results (*e.g*. Bailey, 1964) the distribution of *N*_*t *_is negative binomial with pmf

pn(t;n0)=Pr⁡{Nt=n|N0=n0}=(n−1n0−1)e−λn0t(1−e−λt)n−n0     (2)
 MathType@MTEF@5@5@+=feaafiart1ev1aaatCvAUfKttLearuWrP9MDH5MBPbIqV92AaeXatLxBI9gBaebbnrfifHhDYfgasaacH8akY=wiFfYdH8Gipec8Eeeu0xXdbba9frFj0=OqFfea0dXdd9vqai=hGuQ8kuc9pgc9s8qqaq=dirpe0xb9q8qiLsFr0=vr0=vr0dc8meaabaqaciaacaGaaeqabaqabeGadaaakeaacqWGWbaCdaWgaaWcbaGaemOBa4gabeaakiabcIcaOiabdsha0jabcUda7iabd6gaUnaaBaaaleaacqaIWaamaeqaaOGaeiykaKIaeyypa0JagiiuaaLaeiOCaiNaei4EaSNaemOta40aaSbaaSqaaiabdsha0bqabaGccqGH9aqpcqWGUbGBcqGG8baFcqWGobGtdaWgaaWcbaGaeGimaadabeaakiabg2da9iabd6gaUnaaBaaaleaacqaIWaamaeqaaOGaeiyFa0Naeyypa0ZaaeWaaeaafaqabeGabaaabaGaemOBa4MaeyOeI0IaeGymaedabaGaemOBa42aaSbaaSqaaiabicdaWaqabaGccqGHsislcqaIXaqmaaaacaGLOaGaayzkaaGaemyzau2aaWbaaSqabeaacqGHsisliiGacqWF7oaBcqWGUbGBdaWgaaadbaGaeGimaadabeaaliabdsha0baakiabcIcaOiabigdaXiabgkHiTiabdwgaLnaaCaaaleqabaGaeyOeI0Iae83UdWMaemiDaqhaaOGaeiykaKYaaWbaaSqabeaacqWGUbGBcqGHsislcqWGUbGBdaWgaaadbaGaeGimaadabeaaaaGccaWLjaGaaCzcamaabmaabaGaeGOmaidacaGLOaGaayzkaaaaaa@6DE5@

for *n *= *n*_0_, *n*_0 _+ 1,....

We now consider evolution of genera, and of species within genera, over an epoch between cataclysmic events. Let the time origin be the time of the previous cataclysm, and suppose only a single genus (containing *n*_0 _species) survived that cataclysm. Let *τ *be the time of the succeeding cataclysm. Yule assumed that new genera were formed from old in a process analogous to that of speciation, thereby establishing that the time in existence of any genus would follow a truncated exponential distribution, with parameter equal to the rate at which new genera are formed from old. But it is more realistic to assume that a new genus is formed when a speciation within an existing genus is of such a radical form as to qualify the new species as belonging to a completely new genus. Thus the probabilty of a new genus being formed in an infinitesimal interval (*t*, *t *+ *dt*) should be proportional to *the existing number of species in all existing genera in the family *(and not to the existing number of genera in the family). We let

*K*_*t *_denote the number of genera at time *t*, evolved from the pioneeering *n*_0 _species;

*L*_*t *_denote the number of species at time *t *in all genera, evolved from the pioneeering *n*_0 _species; and

*N*_*t *_denote the number of species in the pioneering genus at time *t*.

We assume that speciations (within a genus) occur at the rate *λ *and new genera are formed from existing species at the rate *γ*. Then to order *o*(*dt*) the following state transitions (of *K*_*t*_, *L*_*t*_, *N*_*t*_) can occur in (*t*, *t *+ *dt*):

(*k*, *l *- 1, *n *- 1) → (*k*, *l*, *n*) with probability *λ*(*n *- 1)*dt*

(*k*, *l *- 1, *n*) → (*k*, *l*, *n*) with probability *λ*(*l *- 1 - *n*)*dt*

(*k *- 1, *l *- 1, *n*) → (*k*, *l*, *n*) with probability *γ*(*l *- 1)*dt*

(*k*, *l*, *n*) → (*k*, *l*, *n*) with probability 1 - (*λ *+ *γ*)*ldt*.

Letting *p*_*k*, *l*, *n*_(*t*) = P(*K*_*t *_= *k*, *L*_*t *_= *l*, *N*_*t *_= *n*), the following differential-difference equations can be established from the above:

ddtpk,l,n(t)=λ(n−1)pk,l−1,n−1(t)+λ(l−1−n)pk,l−1,n(t)+γ(l−1)pk−1,l−1,n(t)−(λ+γ)lpk,l,n(t).     (3)
 MathType@MTEF@5@5@+=feaafiart1ev1aaatCvAUfKttLearuWrP9MDH5MBPbIqV92AaeXatLxBI9gBaebbnrfifHhDYfgasaacH8akY=wiFfYdH8Gipec8Eeeu0xXdbba9frFj0=OqFfea0dXdd9vqai=hGuQ8kuc9pgc9s8qqaq=dirpe0xb9q8qiLsFr0=vr0=vr0dc8meaabaqaciaacaGaaeqabaqabeGadaaakeaafaqabeGadaaabaWaaSaaaeaacqWGKbazaeaacqWGKbazcqWG0baDaaGaemiCaa3aaSbaaSqaaiabdUgaRjabcYcaSiabdYgaSjabcYcaSiabd6gaUbqabaGccqGGOaakcqWG0baDcqGGPaqkaeaacqGH9aqpaeaaiiGacqWF7oaBcqGGOaakcqWGUbGBcqGHsislcqaIXaqmcqGGPaqkcqWGWbaCdaWgaaWcbaGaem4AaSMaeiilaWIaemiBaWMaeyOeI0IaeGymaeJaeiilaWIaemOBa4MaeyOeI0IaeGymaedabeaakiabcIcaOiabdsha0jabcMcaPiabgUcaRiab=T7aSjabcIcaOiabdYgaSjabgkHiTiabigdaXiabgkHiTiabd6gaUjabcMcaPiabdchaWnaaBaaaleaacqWGRbWAcqGGSaalcqWGSbaBcqGHsislcqaIXaqmcqGGSaalcqWGUbGBaeqaaOGaeiikaGIaemiDaqNaeiykaKcabaaabaaabaGaey4kaSIae83SdCMaeiikaGIaemiBaWMaeyOeI0IaeGymaeJaeiykaKIaemiCaa3aaSbaaSqaaiabdUgaRjabgkHiTiabigdaXiabcYcaSiabdYgaSjabgkHiTiabigdaXiabcYcaSiabd6gaUbqabaGccqGGOaakcqWG0baDcqGGPaqkcqGHsislcqGGOaakcqWF7oaBcqGHRaWkcqWFZoWzcqGGPaqkcqWGSbaBcqWGWbaCdaWgaaWcbaGaem4AaSMaeiilaWIaemiBaWMaeiilaWIaemOBa4gabeaakiabcIcaOiabdsha0jabcMcaPiabc6caUaaacaWLjaGaaCzcamaabmaabaGaeG4mamdacaGLOaGaayzkaaaaaa@954A@

Using the generating function

Φ(x,y,z;t)=∑k=1∞∑l=1∞∑n=1∞pk,l,n(t)xkylzn,     (4)
 MathType@MTEF@5@5@+=feaafiart1ev1aaatCvAUfKttLearuWrP9MDH5MBPbIqV92AaeXatLxBI9gBaebbnrfifHhDYfgasaacH8akY=wiFfYdH8Gipec8Eeeu0xXdbba9frFj0=OqFfea0dXdd9vqai=hGuQ8kuc9pgc9s8qqaq=dirpe0xb9q8qiLsFr0=vr0=vr0dc8meaabaqaciaacaGaaeqabaqabeGadaaakeaacqqHMoGrcqGGOaakcqWG4baEcqGGSaalcqWG5bqEcqGGSaalcqWG6bGEcqGG7aWocqWG0baDcqGGPaqkcqGH9aqpdaaeWbqaamaaqahabaWaaabCaeaacqWGWbaCdaWgaaWcbaGaem4AaSMaeiilaWIaemiBaWMaeiilaWIaemOBa4gabeaaaeaacqWGUbGBcqGH9aqpcqaIXaqmaeaacqGHEisPa0GaeyyeIuoakiabcIcaOiabdsha0jabcMcaPiabdIha4naaCaaaleqabaGaem4AaSgaaOGaemyEaK3aaWbaaSqabeaacqWGSbaBaaGccqWG6bGEdaahaaWcbeqaaiabd6gaUbaaaeaacqWGSbaBcqGH9aqpcqaIXaqmaeaacqGHEisPa0GaeyyeIuoaaSqaaiabdUgaRjabg2da9iabigdaXaqaaiabg6HiLcqdcqGHris5aOGaeiilaWIaaCzcaiaaxMaadaqadaqaaiabisda0aGaayjkaiaawMcaaaaa@670D@

multiplying (3) by *x*^*k*^*y*^*l*^*z*^*n *^and summing yields the following partial differential equation

Φ_*t *_= *y*(*λy *+ *γxy *- (*λ *+ *γ*)) Φ_*y *_+ *λyz*(*z *- 1) Φ_*z*_,     (5)

which can be solved by the method of characteristics (*e.g*. Bailey,[6]) with initial condition *ϕ*(*x*, *y*, *z*; 0) = xyn0zn0
 MathType@MTEF@5@5@+=feaafiart1ev1aaatCvAUfKttLearuWrP9MDH5MBPbIqV92AaeXatLxBI9gBaebbnrfifHhDYfgasaacH8akY=wiFfYdH8Gipec8Eeeu0xXdbba9frFj0=OqFfea0dXdd9vqai=hGuQ8kuc9pgc9s8qqaq=dirpe0xb9q8qiLsFr0=vr0=vr0dc8meaabaqaciaacaGaaeqabaqabeGadaaakeaacqWG4baEcqWG5bqEdaahaaWcbeqaaiabd6gaUnaaBaaameaacqaIWaamaeqaaaaakiabdQha6naaCaaaleqabaGaemOBa42aaSbaaWqaaiabicdaWaqabaaaaaaa@3681@. From the solution the generating functions of *K*_*t*_, *L*_*t *_and *N*_*t *_can be derived. They are

ΦK(x,t)=E(xKt)=x{p(t)1−x[1−p(t)]}n0,     (6)
 MathType@MTEF@5@5@+=feaafiart1ev1aaatCvAUfKttLearuWrP9MDH5MBPbIqV92AaeXatLxBI9gBaebbnrfifHhDYfgasaacH8akY=wiFfYdH8Gipec8Eeeu0xXdbba9frFj0=OqFfea0dXdd9vqai=hGuQ8kuc9pgc9s8qqaq=dirpe0xb9q8qiLsFr0=vr0=vr0dc8meaabaqaciaacaGaaeqabaqabeGadaaakeaacqqHMoGrdaWgaaWcbaGaem4saSeabeaakiabcIcaOiabdIha4jabcYcaSiabdsha0jabcMcaPiabg2da9iabdweafjabcIcaOiabdIha4naaCaaaleqabaGaem4saS0aaSbaaWqaaiabdsha0bqabaaaaOGaeiykaKIaeyypa0JaemiEaG3aaiWabeaadaWcaaqaaiabdchaWjabcIcaOiabdsha0jabcMcaPaqaaiabigdaXiabgkHiTiabdIha4jabcUfaBjabigdaXiabgkHiTiabdchaWjabcIcaOiabdsha0jabcMcaPiabc2faDbaaaiaawUhacaGL9baadaahaaWcbeqaaiabd6gaUnaaBaaameaacqaIWaamaeqaaaaakiabcYcaSiaaxMaacaWLjaWaaeWaaeaacqaI2aGnaiaawIcacaGLPaaaaaa@5A19@

ΦL(y,t)=E(yLt)={ye−(λ+γ)t1−y[1−e−(λ+γ)t]}n0,     (7)
 MathType@MTEF@5@5@+=feaafiart1ev1aaatCvAUfKttLearuWrP9MDH5MBPbIqV92AaeXatLxBI9gBaebbnrfifHhDYfgasaacH8akY=wiFfYdH8Gipec8Eeeu0xXdbba9frFj0=OqFfea0dXdd9vqai=hGuQ8kuc9pgc9s8qqaq=dirpe0xb9q8qiLsFr0=vr0=vr0dc8meaabaqaciaacaGaaeqabaqabeGadaaakeaacqqHMoGrdaWgaaWcbaGaemitaWeabeaakiabcIcaOiabdMha5jabcYcaSiabdsha0jabcMcaPiabg2da9iabdweafjabcIcaOiabdMha5naaCaaaleqabaGaemitaW0aaSbaaWqaaiabdsha0bqabaaaaOGaeiykaKIaeyypa0ZaaiWabeaadaWcaaqaaiabdMha5jabdwgaLnaaCaaaleqabaGaeyOeI0IaeiikaGccciGae83UdWMaey4kaSIae83SdCMaeiykaKIaemiDaqhaaaGcbaGaeGymaeJaeyOeI0IaemyEaKNaei4waSLaeGymaeJaeyOeI0Iaemyzau2aaWbaaSqabeaacqGHsislcqGGOaakcqWF7oaBcqGHRaWkcqWFZoWzcqGGPaqkcqWG0baDaaGccqGGDbqxaaaacaGL7bGaayzFaaWaaWbaaSqabeaacqWGUbGBdaWgaaadbaGaeGimaadabeaaaaGccqGGSaalcaWLjaGaaCzcamaabmaabaGaeG4naCdacaGLOaGaayzkaaaaaa@64B5@

ΦN(z,t)=E(zNt)={ze−λt1−z(1−e−λt)}n0,     (8)
 MathType@MTEF@5@5@+=feaafiart1ev1aaatCvAUfKttLearuWrP9MDH5MBPbIqV92AaeXatLxBI9gBaebbnrfifHhDYfgasaacH8akY=wiFfYdH8Gipec8Eeeu0xXdbba9frFj0=OqFfea0dXdd9vqai=hGuQ8kuc9pgc9s8qqaq=dirpe0xb9q8qiLsFr0=vr0=vr0dc8meaabaqaciaacaGaaeqabaqabeGadaaakeaacqqHMoGrdaWgaaWcbaGaemOta4eabeaakiabcIcaOiabdQha6jabcYcaSiabdsha0jabcMcaPiabg2da9iabdweafjabcIcaOiabdQha6naaCaaaleqabaGaemOta40aaSbaaWqaaiabdsha0bqabaaaaOGaeiykaKIaeyypa0ZaaiWabeaadaWcaaqaaiabdQha6jabdwgaLnaaCaaaleqabaGaeyOeI0ccciGae83UdWMaemiDaqhaaaGcbaGaeGymaeJaeyOeI0IaemOEaONaeiikaGIaeGymaeJaeyOeI0Iaemyzau2aaWbaaSqabeaacqGHsislcqWF7oaBcqWG0baDaaGccqGGPaqkaaaacaGL7bGaayzFaaWaaWbaaSqabeaacqWGUbGBdaWgaaadbaGaeGimaadabeaaaaGccqGGSaalcaWLjaGaaCzcamaabmaabaGaeGioaGdacaGLOaGaayzkaaaaaa@5B8D@

where

p(t)=(λ+γ)e−(λ+γ)tγ+λe−(λ+γ)t.     (9)
 MathType@MTEF@5@5@+=feaafiart1ev1aaatCvAUfKttLearuWrP9MDH5MBPbIqV92AaeXatLxBI9gBaebbnrfifHhDYfgasaacH8akY=wiFfYdH8Gipec8Eeeu0xXdbba9frFj0=OqFfea0dXdd9vqai=hGuQ8kuc9pgc9s8qqaq=dirpe0xb9q8qiLsFr0=vr0=vr0dc8meaabaqaciaacaGaaeqabaqabeGadaaakeaacqWGWbaCcqGGOaakcqWG0baDcqGGPaqkcqGH9aqpdaWcaaqaaiabcIcaOGGaciab=T7aSjabgUcaRiab=n7aNjabcMcaPiabdwgaLnaaCaaaleqabaGaeyOeI0IaeiikaGIae83UdWMaey4kaSIae83SdCMaeiykaKIaemiDaqhaaaGcbaGae83SdCMaey4kaSIae83UdWMaemyzau2aaWbaaSqabeaacqGHsislcqGGOaakcqWF7oaBcqGHRaWkcqWFZoWzcqGGPaqkcqWG0baDaaaaaOGaeiOla4IaaCzcaiaaxMaadaqadaqaaiabiMda5aGaayjkaiaawMcaaaaa@54BD@

From this it is clear that both the total number of species, *L*_*t*_, and the number of species in the pioneering genus, *N*_*t*_, have negative binomial distributions (with parameters *n*_0 _and *e*^-(*λ*+ *γ*)*t *^and n_0 _and *e*^-*λt *^respectively); while the number of genera *K*_*t *_has a distribution related to the negative binomial – precisely *K*_*t *_+ *n*_0 _- 1 has a negative binomial distribution with parameters *n*_0 _and *p*(*t*). The expected number of genera at time *t *is

E(Kt)=1+n0γλ+γ[e(λ+γ)t−1].     (10)
 MathType@MTEF@5@5@+=feaafiart1ev1aaatCvAUfKttLearuWrP9MDH5MBPbIqV92AaeXatLxBI9gBaebbnrfifHhDYfgasaacH8akY=wiFfYdH8Gipec8Eeeu0xXdbba9frFj0=OqFfea0dXdd9vqai=hGuQ8kuc9pgc9s8qqaq=dirpe0xb9q8qiLsFr0=vr0=vr0dc8meaabaqaciaacaGaaeqabaqabeGadaaakeaaieaacqWFfbqrcqGGOaakcqWGlbWsdaWgaaWcbaGaemiDaqhabeaakiabcMcaPiabg2da9iabigdaXiabgUcaRmaalaaabaGaemOBa42aaSbaaSqaaiabicdaWaqabaacciGccqGFZoWzaeaacqGF7oaBcqGHRaWkcqGFZoWzaaWaamWaaeaacqWGLbqzdaahaaWcbeqaaiabcIcaOiab+T7aSjabgUcaRiab+n7aNjabcMcaPiabdsha0baakiabgkHiTiabigdaXaGaay5waiaaw2faaiabc6caUiaaxMaacaWLjaWaaeWaaeaacqaIXaqmcqaIWaamaiaawIcacaGLPaaaaaa@4FC7@

It can be shown (see Appendix) that the times of formation of derived genera constitute an *order statistic process*. This means that they can be considered as the order statisics of a collection of independent, identically distributed (iid) random variables. From this it is shown that at any fixed time *τ*, the times *t*_1_, *t*_2_,...,*t*_*k *_that the derived genera have been in existence are iid random variables with probability density function (pdf)

fk(t)=(λ+γ)e−(λ+γ)t1−e−(λ+γ)τ,0<t<τ.     (11)
 MathType@MTEF@5@5@+=feaafiart1ev1aaatCvAUfKttLearuWrP9MDH5MBPbIqV92AaeXatLxBI9gBaebbnrfifHhDYfgasaacH8akY=wiFfYdH8Gipec8Eeeu0xXdbba9frFj0=OqFfea0dXdd9vqai=hGuQ8kuc9pgc9s8qqaq=dirpe0xb9q8qiLsFr0=vr0=vr0dc8meaabaqaciaacaGaaeqabaqabeGadaaakeaafaqabeqacaaabaGaemOzay2aaSbaaSqaaiabdUgaRbqabaGccqGGOaakcqWG0baDcqGGPaqkcqGH9aqpdaWcaaqaaiabcIcaOGGaciab=T7aSjabgUcaRiab=n7aNjabcMcaPiabdwgaLnaaCaaaleqabaGaeyOeI0IaeiikaGIae83UdWMaey4kaSIae83SdCMaeiykaKIaemiDaqhaaaGcbaGaeGymaeJaeyOeI0Iaemyzau2aaWbaaSqabeaacqGHsislcqGGOaakcqWF7oaBcqGHRaWkcqWFZoWzcqGGPaqkcqWFepaDaaaaaOGaeiilaWcabaGaeGimaaJaeyipaWJaemiDaqNaeyipaWJae8hXdqhaaiabc6caUiaaxMaacaWLjaWaaeWaaeaacqaIXaqmcqaIXaqmaiaawIcacaGLPaaaaaa@5C2B@

By summing (3) over *k *and *l *one can show that *N*_*t *_is a pure birth process with birthrate *λ*; and by summing over *k *and *n *that *L*_*t *_is a pure birth process with birthrate *λ *+ *γ*. From the fact that a pure birth process is an order statistic process it can be shown (see Appendix) that at time *τ *the times since establishment of all non-pioneering species in the pioneering *genus *are independently distributed random variables, with a truncated exponential distribution with pdf

fN(t)=λ e−λt1−e−λτ,0<t<τ;     (12)
 MathType@MTEF@5@5@+=feaafiart1ev1aaatCvAUfKttLearuWrP9MDH5MBPbIqV92AaeXatLxBI9gBaebbnrfifHhDYfgasaacH8akY=wiFfYdH8Gipec8Eeeu0xXdbba9frFj0=OqFfea0dXdd9vqai=hGuQ8kuc9pgc9s8qqaq=dirpe0xb9q8qiLsFr0=vr0=vr0dc8meaabaqaciaacaGaaeqabaqabeGadaaakeaafaqabeqacaaabaGaemOzay2aaSbaaSqaaiabd6eaobqabaGccqGGOaakcqWG0baDcqGGPaqkcqGH9aqpdaWcaaqaaGGaciab=T7aSjabbccaGiabdwgaLnaaCaaaleqabaGaeyOeI0Iae83UdWMaemiDaqhaaaGcbaGaeGymaeJaeyOeI0Iaemyzau2aaWbaaSqabeaacqGHsislcqWF7oaBcqWFepaDaaaaaOGaeiilaWcabaGaeGimaaJaeyipaWJaemiDaqNaeyipaWJae8hXdqNaei4oaSdaaiaaxMaacaWLjaWaaeWaaeaacqaIXaqmcqaIYaGmaiaawIcacaGLPaaaaaa@5032@

and that the times since establishment of all non-pioneering species in the pioneering *family *are independently distributed random variables, with a truncated exponential distribution with pdf

fL(t)=(λ+γ)e−(λ+γ)t1−e−(λ+γ)τ,0<t<τ.     (13)
 MathType@MTEF@5@5@+=feaafiart1ev1aaatCvAUfKttLearuWrP9MDH5MBPbIqV92AaeXatLxBI9gBaebbnrfifHhDYfgasaacH8akY=wiFfYdH8Gipec8Eeeu0xXdbba9frFj0=OqFfea0dXdd9vqai=hGuQ8kuc9pgc9s8qqaq=dirpe0xb9q8qiLsFr0=vr0=vr0dc8meaabaqaciaacaGaaeqabaqabeGadaaakeaafaqabeqacaaabaGaemOzay2aaSbaaSqaaiabdYeambqabaGccqGGOaakcqWG0baDcqGGPaqkcqGH9aqpdaWcaaqaaiabcIcaOGGaciab=T7aSjabgUcaRiab=n7aNjabcMcaPiabdwgaLnaaCaaaleqabaGaeyOeI0IaeiikaGIae83UdWMaey4kaSIae83SdCMaeiykaKIaemiDaqhaaaGcbaGaeGymaeJaeyOeI0Iaemyzau2aaWbaaSqabeaacqGHsislcqGGOaakcqWF7oaBcqGHRaWkcqWFZoWzcqGGPaqkcqWFepaDaaaaaOGaeiilaWcabaGaeGimaaJaeyipaWJaemiDaqNaeyipaWJae8hXdqhaaiabc6caUiaaxMaacaWLjaWaaeWaaeaacqaIXaqmcqaIZaWmaiaawIcacaGLPaaaaaa@5BF1@

Note the fact that *f*_*L*_(*t*) ≡ *f*_*K*_(*t*) *i.e*. the marginal distribution of the time since establishment of a derived genus in the family is the same as that of a derived species in the family.

Consider now the case when *τ *is the time of the first cataclysm since the appearance of the pioneering genus. The size distribution of all derived (non-pioneering) genera at the time of the cataclysm can be obtained by integrating the geometric pmf *p*_*n*_(*t*; 1) in (1) with respect to the truncated exponential distribution *f*_*K*_(*t*) between 0 and *τ*. This yields the pmf

qnderiv=∫0τpn(t;1)fK(t)dt=1+γ/λ1+e−(λ+γ)τ[B(2+γ/λ,n)−Be−λτ(2+γ/λ,n)],     (14)
 MathType@MTEF@5@5@+=feaafiart1ev1aaatCvAUfKttLearuWrP9MDH5MBPbIqV92AaeXatLxBI9gBaebbnrfifHhDYfgasaacH8akY=wiFfYdH8Gipec8Eeeu0xXdbba9frFj0=OqFfea0dXdd9vqai=hGuQ8kuc9pgc9s8qqaq=dirpe0xb9q8qiLsFr0=vr0=vr0dc8meaabaqaciaacaGaaeqabaqabeGadaaakeaafaqaaeGadaaabaGaemyCae3aa0baaSqaaiabd6gaUbqaaGqaaiab=rgaKjab=vgaLjab=jhaYjab=LgaPjab=zha2baaaOqaaiabg2da9aqaamaapedabaGaemiCaa3aaSbaaSqaaiabd6gaUbqabaGccqGGOaakcqWG0baDcqGG7aWocqaIXaqmcqGGPaqkcqWGMbGzdaWgaaWcbaGaem4saSeabeaakiabcIcaOiabdsha0jabcMcaPiabdsgaKjabdsha0bWcbaGaeGimaadabaacciGae4hXdqhaniabgUIiYdaakeaaaeaacqGH9aqpaeaadaWcaaqaaiabigdaXiabgUcaRiab+n7aNjabc+caViab+T7aSbqaaiabigdaXiabgUcaRiabdwgaLnaaCaaaleqabaGaeyOeI0IaeiikaGIae43UdWMaey4kaSIae43SdCMaeiykaKIae4hXdqhaaaaakiabcUfaBjabdkeacjabcIcaOiabikdaYiabgUcaRiab+n7aNjabc+caViab+T7aSjabcYcaSiabd6gaUjabcMcaPiabgkHiTiabdkeacnaaBaaaleaacqWGLbqzdaahaaadbeqaaiabgkHiTiab+T7aSjab+r8a0baaaSqabaGccqGGOaakcqaIYaGmcqGHRaWkcqGFZoWzcqGGVaWlcqGF7oaBcqGGSaalcqWGUbGBcqGGPaqkcqGGDbqxcqGGSaalaaGaaCzcaiaaxMaadaqadaqaaiabigdaXiabisda0aGaayjkaiaawMcaaaaa@856F@

where

B(a,b)=Γ(a)Γ(b)Γ(a+b),Bx(a,b)=∫0xza−1(1−z)b−1dz
 MathType@MTEF@5@5@+=feaafiart1ev1aaatCvAUfKttLearuWrP9MDH5MBPbIqV92AaeXatLxBI9gBaebbnrfifHhDYfgasaacH8akY=wiFfYdH8Gipec8Eeeu0xXdbba9frFj0=OqFfea0dXdd9vqai=hGuQ8kuc9pgc9s8qqaq=dirpe0xb9q8qiLsFr0=vr0=vr0dc8meaabaqaciaacaGaaeqabaqabeGadaaakeaafaqabeqacaaabaGaemOqaiKaeiikaGIaemyyaeMaeiilaWIaemOyaiMaeiykaKIaeyypa0ZaaSaaaeaacqqHtoWrcqGGOaakcqWGHbqycqGGPaqkcqqHtoWrcqGGOaakcqWGIbGycqGGPaqkaeaacqqHtoWrcqGGOaakcqWGHbqycqGHRaWkcqWGIbGycqGGPaqkaaGaeiilaWcabaGaemOqai0aaSbaaSqaaiabdIha4bqabaGccqGGOaakcqWGHbqycqGGSaalcqWGIbGycqGGPaqkcqGH9aqpdaWdXaqaaiabdQha6naaCaaaleqabaGaemyyaeMaeyOeI0IaeGymaedaaOGaeiikaGIaeGymaeJaeyOeI0IaemOEaONaeiykaKYaaWbaaSqabeaacqWGIbGycqGHsislcqaIXaqmaaGccqWGKbazcqWG6bGEaSqaaiabicdaWaqaaiabdIha4bqdcqGHRiI8aaaaaaa@61D9@

are the *beta function *and *incomplete beta functions*, respectively. Alternatively the term in square brackets can be expressed in terms of the cumulative distribution function (cdf) *F*(*x*; *a*, *b*) of the *beta distribution *with parameters *a *and *b *leading to

qnderiv=(1+γ/λ)B(2+γ/λ,n)1−e−(λ+γ)τ[1−F(e−λτ;2+γ/λ,n)].     (15)
 MathType@MTEF@5@5@+=feaafiart1ev1aaatCvAUfKttLearuWrP9MDH5MBPbIqV92AaeXatLxBI9gBaebbnrfifHhDYfgasaacH8akY=wiFfYdH8Gipec8Eeeu0xXdbba9frFj0=OqFfea0dXdd9vqai=hGuQ8kuc9pgc9s8qqaq=dirpe0xb9q8qiLsFr0=vr0=vr0dc8meaabaqaciaacaGaaeqabaqabeGadaaakeaacqWGXbqCdaqhaaWcbaGaemOBa4gabaacbaGae8hzaqMae8xzauMae8NCaiNae8xAaKMae8NDayhaaOGaeyypa0ZaaSaaaeaacqGGOaakcqaIXaqmcqGHRaWkiiGacqGFZoWzcqGGVaWlcqGF7oaBcqGGPaqkcqWGcbGqcqGGOaakcqaIYaGmcqGHRaWkcqGFZoWzcqGGVaWlcqGF7oaBcqGGSaalcqWGUbGBcqGGPaqkaeaacqaIXaqmcqGHsislcqWGLbqzdaahaaWcbeqaaiabgkHiTiabcIcaOiab+T7aSjabgUcaRiab+n7aNjabcMcaPiab+r8a0baaaaGcdaWadaqaaiabigdaXiabgkHiTiabdAeagjabcIcaOiabdwgaLnaaCaaaleqabaGaeyOeI0Iae43UdWMae4hXdqhaaOGaei4oaSJaeGOmaiJaey4kaSIae43SdCMaei4la8Iae43UdWMaeiilaWIaemOBa4MaeiykaKcacaGLBbGaayzxaaGaeiOla4IaaCzcaiaaxMaadaqadaqaaiabigdaXiabiwda1aGaayjkaiaawMcaaaaa@71C6@

This can be readily computed using standard statistical software.

The distribution of the size of the pioneering genus at time *τ *has pmf qnpion
 MathType@MTEF@5@5@+=feaafiart1ev1aaatCvAUfKttLearuWrP9MDH5MBPbIqV92AaeXatLxBI9gBaebbnrfifHhDYfgasaacH8akY=wiFfYdH8Gipec8Eeeu0xXdbba9frFj0=OqFfea0dXdd9vqai=hGuQ8kuc9pgc9s8qqaq=dirpe0xb9q8qiLsFr0=vr0=vr0dc8meaabaqaciaacaGaaeqabaqabeGadaaakeaacqWGXbqCdaqhaaWcbaGaemOBa4gabaacbaGae8hCaaNae8xAaKMae83Ba8Mae8NBa4gaaaaa@3532@ = *p*_*n*_(*τ*; *n*_0_) where *p*_*n *_is negative binomial pmf given by (2). The distribution of the size of all existing genera at time *τ *is simply a mixture of qnpion
 MathType@MTEF@5@5@+=feaafiart1ev1aaatCvAUfKttLearuWrP9MDH5MBPbIqV92AaeXatLxBI9gBaebbnrfifHhDYfgasaacH8akY=wiFfYdH8Gipec8Eeeu0xXdbba9frFj0=OqFfea0dXdd9vqai=hGuQ8kuc9pgc9s8qqaq=dirpe0xb9q8qiLsFr0=vr0=vr0dc8meaabaqaciaacaGaaeqabaqabeGadaaakeaacqWGXbqCdaqhaaWcbaGaemOBa4gabaacbaGae8hCaaNae8xAaKMae83Ba8Mae8NBa4gaaaaa@3532@ and qnderiv
 MathType@MTEF@5@5@+=feaafiart1ev1aaatCvAUfKttLearuWrP9MDH5MBPbIqV92AaeXatLxBI9gBaebbnrfifHhDYfgasaacH8akY=wiFfYdH8Gipec8Eeeu0xXdbba9frFj0=OqFfea0dXdd9vqai=hGuQ8kuc9pgc9s8qqaq=dirpe0xb9q8qiLsFr0=vr0=vr0dc8meaabaqaciaacaGaaeqabaqabeGadaaakeaacqWGXbqCdaqhaaWcbaGaemOBa4gabaacbaGae8hzaqMae8xzauMae8NCaiNae8xAaKMae8NDayhaaaaa@367F@. Precisely

qn=πK(τ)qnpion+[1−πK(τ)]qnderiv,     (16)
 MathType@MTEF@5@5@+=feaafiart1ev1aaatCvAUfKttLearuWrP9MDH5MBPbIqV92AaeXatLxBI9gBaebbnrfifHhDYfgasaacH8akY=wiFfYdH8Gipec8Eeeu0xXdbba9frFj0=OqFfea0dXdd9vqai=hGuQ8kuc9pgc9s8qqaq=dirpe0xb9q8qiLsFr0=vr0=vr0dc8meaabaqaciaacaGaaeqabaqabeGadaaakeaacqWGXbqCdaWgaaWcbaGaemOBa4gabeaakiabg2da9GGaciab=b8aWnaaBaaaleaacqWGlbWsaeqaaOGaeiikaGIae8hXdqNaeiykaKIaemyCae3aa0baaSqaaiabd6gaUbqaaGqaaiab+bhaWjab+LgaPjab+9gaVjab+5gaUbaakiabgUcaRiabcUfaBjabigdaXiabgkHiTiab=b8aWnaaBaaaleaacqWGlbWsaeqaaOGaeiikaGIae8hXdqNaeiykaKIaeiyxa0LaemyCae3aa0baaSqaaiabd6gaUbqaaiab+rgaKjab+vgaLjab+jhaYjab+LgaPjab+zha2baakiabcYcaSiaaxMaacaWLjaWaaeWaaeaacqaIXaqmcqaI2aGnaiaawIcacaGLPaaaaaa@5AF3@

where *π*_*K*_(*τ*) is the probability that a genus in existence at time *τ *is the pioneering genus, *i.e*.

πK(τ)=E(1Kτ)=∫01ΦK(s,τ)sds,     (17)
 MathType@MTEF@5@5@+=feaafiart1ev1aaatCvAUfKttLearuWrP9MDH5MBPbIqV92AaeXatLxBI9gBaebbnrfifHhDYfgasaacH8akY=wiFfYdH8Gipec8Eeeu0xXdbba9frFj0=OqFfea0dXdd9vqai=hGuQ8kuc9pgc9s8qqaq=dirpe0xb9q8qiLsFr0=vr0=vr0dc8meaabaqaciaacaGaaeqabaqabeGadaaakeaaiiGacqWFapaCdaWgaaWcbaGaem4saSeabeaakiabcIcaOiab=r8a0jabcMcaPiabg2da9Gqaaiab+veafnaabmaabaWaaSaaaeaacqaIXaqmaeaacqWGlbWsdaWgaaWcbaGae8hXdqhabeaaaaaakiaawIcacaGLPaaacqGH9aqpdaWdXaqaamaalaaabaGaeuOPdy0aaSbaaSqaaiabdUealbqabaGccqGGOaakcqWGZbWCcqGGSaalcqWFepaDcqGGPaqkaeaacqWGZbWCaaaaleaacqaIWaamaeaacqaIXaqma0Gaey4kIipakiabdsgaKjabdohaZjabcYcaSiaaxMaacaWLjaWaaeWaaeaacqaIXaqmcqaI3aWnaiaawIcacaGLPaaaaaa@5271@

which can be evaluated as

πK(τ)=(λ+γ)e−(λ+γ)τγ[1−e−(λ+γ)τ]log⁡(γ+λe−(λ+γ)τ(λ+γ)e−(λ+γ)τ).     (18)
 MathType@MTEF@5@5@+=feaafiart1ev1aaatCvAUfKttLearuWrP9MDH5MBPbIqV92AaeXatLxBI9gBaebbnrfifHhDYfgasaacH8akY=wiFfYdH8Gipec8Eeeu0xXdbba9frFj0=OqFfea0dXdd9vqai=hGuQ8kuc9pgc9s8qqaq=dirpe0xb9q8qiLsFr0=vr0=vr0dc8meaabaqaciaacaGaaeqabaqabeGadaaakeaaiiGacqWFapaCdaWgaaWcbaGaem4saSeabeaakiabcIcaOiab=r8a0jabcMcaPiabg2da9maalaaabaGaeiikaGIae83UdWMaey4kaSIae83SdCMaeiykaKIaemyzau2aaWbaaSqabeaacqGHsislcqGGOaakcqWF7oaBcqGHRaWkcqWFZoWzcqGGPaqkcqWFepaDaaaakeaacqWFZoWzcqGGBbWwcqaIXaqmcqGHsislcqWGLbqzdaahaaWcbeqaaiabgkHiTiabcIcaOiab=T7aSjabgUcaRiab=n7aNjabcMcaPiab=r8a0baakiabc2faDbaacyGGSbaBcqGGVbWBcqGGNbWzdaqadaqaamaalaaabaGae83SdCMaey4kaSIae83UdWMaemyzau2aaWbaaSqabeaacqGHsislcqGGOaakcqWF7oaBcqGHRaWkcqWFZoWzcqGGPaqkcqWFepaDaaaakeaacqGGOaakcqWF7oaBcqGHRaWkcqWFZoWzcqGGPaqkcqWGLbqzdaahaaWcbeqaaiabgkHiTiabcIcaOiab=T7aSjabgUcaRiab=n7aNjabcMcaPiab=r8a0baaaaaakiaawIcacaGLPaaacqGGUaGlcaWLjaGaaCzcamaabmaabaGaeGymaeJaeGioaGdacaGLOaGaayzkaaaaaa@7E0F@

Note that as *τ *→ ∞, *π*_*K*_(*τ*) → 0 and

qn→(γ/λ+1)Γ(γ/λ+2)Γ(n)Γ(γ/λ+n+2).     (19)
 MathType@MTEF@5@5@+=feaafiart1ev1aaatCvAUfKttLearuWrP9MDH5MBPbIqV92AaeXatLxBI9gBaebbnrfifHhDYfgasaacH8akY=wiFfYdH8Gipec8Eeeu0xXdbba9frFj0=OqFfea0dXdd9vqai=hGuQ8kuc9pgc9s8qqaq=dirpe0xb9q8qiLsFr0=vr0=vr0dc8meaabaqaciaacaGaaeqabaqabeGadaaakeaacqWGXbqCdaWgaaWcbaGaemOBa4gabeaakiabgkziUoaalaaabaGaeiikaGccciGae83SdCMaei4la8Iae83UdWMaey4kaSIaeGymaeJaeiykaKIaeu4KdCKaeiikaGIae83SdCMaei4la8Iae83UdWMaey4kaSIaeGOmaiJaeiykaKIaeu4KdCKaeiikaGIaemOBa4MaeiykaKcabaGaeu4KdCKaeiikaGIae83SdCMaei4la8Iae83UdWMaey4kaSIaemOBa4Maey4kaSIaeGOmaiJaeiykaKcaaiabc6caUiaaxMaacaWLjaWaaeWaaeaacqaIXaqmcqaI5aqoaiaawIcacaGLPaaaaaa@5827@

This distribution was obtained by Yule[1] and is now known as the *Yule distribution*; for this distribution *q*_*n *_behaves asymptotically like a power-law, *i.e*.,

*q*_*n *_~ (*γ*/*λ *+ 1)*Γ*(*γ*/*λ *+ 2) × *n*^-(2 + *γ*/*λ*)^

as *n *→ ∞, yielding the asymptotic straight line when *q*_*n *_is plotted against *n *on logarithmic axes. We note in passing that setting *γ *= 0 in (19) does *not *yield the size distribution (as *τ *→ ∞) of a single genus, since when *γ *= 0, *π*_*K *_≡ 1. In this case *N*_*τ *_→ ∞ with probability one.

**Figure 1:**
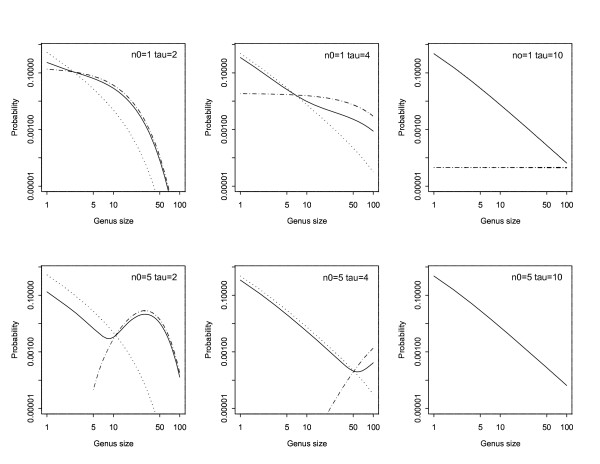
Logarithmic plots (both scales logarithmic) of the size distribution of genera, assuming only cataclysmic extinctions. The top row corresponds to *n*_0 _= 1 and the bottom row to *n*_0 _= 5. The three columns (from left to right) correspond to *τ *= 2,4 and 10. In all cases *λ *= 1 and *γ *= 0.1. For the sake of display the points of the probability mass function have been joined by lines:- dotted for derived genera; dot-dash for the pioneering genus and solid for the mixed distribution of all genera. The distribution of the pioneering genus (dot-dash) does not appear in the lower right-hand panel because the pmf assumes values less than 0.0001 for all sizes up to 100. In consequence the mixed distribution (solid line) is overlaid on that of derived genera (dotted line). Similarly in the upper right-hand panel the dotted and solid lines are overlaid.

Figure 1 shows the size distribution of pioneering and derived genera, along with the mixed distribution of all genera, calculated from the above formulae, for different values of *n*_0 _and *τ*. They show how the results of Yule [1] need to be modified to take into account the effects of: (a) the evolution of new genera ; (b) pioneering genera of size (*n*_0_) greater than one; and (c) the time, *τ*, until cataclysmic extinction. Large values of *τ *(right-hand panels), resulting in straight-line plots on the log-log scale, correspond most closely to the situation considered initially by Yule. In this case approximate power-law (fractal) distributions occur. The deviations from such a power-law distribution are greatest when cataclysmic extinction occurs earlier (smaller *τ*) and when the number of species in the pioneering genus (*n*_0_) differs greatly from one (lower panels). The distribution of derived genera (dotted lines) is unaffected by the initial size (*n*_0_) of the pioneering genus. However the overall size distribution is affected (especially at values immediately above *n*_0_) because of the fact that the pioneering genus size has support on {*n*_0_, *n*_0 _+ 1,...} while that of derived genera is on {1, 2,...}. This effect becomes less important when a long time elapses before the cataclysmic extinction event (because when *τ *is large, *π*_*K*_(*τ*) is small–derived genera will in probability outnumber the pioneering one).

## Background extinctions only

In this section we consider the size distribution of a fossil genus, starting with a single species (the case of a genus beginning with *n*_0 _species is considered later in this section), subject to speciations at rate *λ *and background (individual) extinctions occurring independently and at random, at rate *μ*.

Thus *N*_*t*_, the number of species alive *t *time units after the origin of the genus, follows a homogeneous birth and death process. Let *M*_*t *_denote the total number of species in the genus that have existed by time *t *(*i.e*. *M*_*t *_= 1 + number of speciations). The size of an extinct genus is a random variable *M*_*T*_, where *T *itself is a random variable, denoting the time of extinction. Since no speciations can occur in a genus once it is extinct, we have that for *t *≥ *T*, *M*_*t *_≡ *M*_*T*_. However *T *may not be finite (*N*_*t *_> 0 for all *t*). Thus finding the distribution of the size of an extinct genus will involve conditioning on *T *< ∞ (or *N*_∞ _= 0). Clearly it is given by the distribution of *M*_∞ _conditional on *N*_∞ _= 0.

Now let

*p*_*m*, *n*_(*t*) = Pr(*M*_*t *_= *m*, *N*_*t *_= *n*).     (20)

It was shown by Kendall[7] that *p*_*m*, *n *_satisfies the differential-difference equations

ddtpm,n(t)=−(λ+μ)npm,n(t)+λ(n−1)pm−1,n−1(t)+μ(n+1)pm,n+1(t)     (21)
 MathType@MTEF@5@5@+=feaafiart1ev1aaatCvAUfKttLearuWrP9MDH5MBPbIqV92AaeXatLxBI9gBaebbnrfifHhDYfgasaacH8akY=wiFfYdH8Gipec8Eeeu0xXdbba9frFj0=OqFfea0dXdd9vqai=hGuQ8kuc9pgc9s8qqaq=dirpe0xb9q8qiLsFr0=vr0=vr0dc8meaabaqaciaacaGaaeqabaqabeGadaaakeaadaWcaaqaaiabdsgaKbqaaiabdsgaKjabdsha0baacqWGWbaCdaWgaaWcbaGaemyBa0MaeiilaWIaemOBa4gabeaakiabcIcaOiabdsha0jabcMcaPiabg2da9iabgkHiTiabcIcaOGGaciab=T7aSjabgUcaRiab=X7aTjabcMcaPiabd6gaUjabdchaWnaaBaaaleaacqWGTbqBcqGGSaalcqWGUbGBaeqaaOGaeiikaGIaemiDaqNaeiykaKIaey4kaSIae83UdWMaeiikaGIaemOBa4MaeyOeI0IaeGymaeJaeiykaKIaemiCaa3aaSbaaSqaaiabd2gaTjabgkHiTiabigdaXiabcYcaSiabd6gaUjabgkHiTiabigdaXaqabaGccqGGOaakcqWG0baDcqGGPaqkcqGHRaWkcqWF8oqBcqGGOaakcqWGUbGBcqGHRaWkcqaIXaqmcqGGPaqkcqWGWbaCdaWgaaWcbaGaemyBa0MaeiilaWIaemOBa4Maey4kaSIaeGymaedabeaakiabcIcaOiabdsha0jabcMcaPiaaxMaacaWLjaWaaeWaaeaacqaIYaGmcqaIXaqmaiaawIcacaGLPaaaaaa@750B@

with initial condition

*p*_*m*, *n*_(0) = 1 if *m *= *n *= 1; *p*_*m*, *n*_(0) = 0 otherwise.

Let

Ψ(s,z;t)=∑m=1∞∑n=0∞pm,n(t)smzn     (22)
 MathType@MTEF@5@5@+=feaafiart1ev1aaatCvAUfKttLearuWrP9MDH5MBPbIqV92AaeXatLxBI9gBaebbnrfifHhDYfgasaacH8akY=wiFfYdH8Gipec8Eeeu0xXdbba9frFj0=OqFfea0dXdd9vqai=hGuQ8kuc9pgc9s8qqaq=dirpe0xb9q8qiLsFr0=vr0=vr0dc8meaabaqaciaacaGaaeqabaqabeGadaaakeaacqqHOoqwcqGGOaakcqWGZbWCcqGGSaalcqWG6bGEcqGG7aWocqWG0baDcqGGPaqkcqGH9aqpdaaeWbqaamaaqahabaGaemiCaa3aaSbaaSqaaiabd2gaTjabcYcaSiabd6gaUbqabaGccqGGOaakcqWG0baDcqGGPaqkcqWGZbWCdaahaaWcbeqaaiabd2gaTbaakiabdQha6naaCaaaleqabaGaemOBa4gaaaqaaiabd6gaUjabg2da9iabicdaWaqaaiabg6HiLcqdcqGHris5aaWcbaGaemyBa0Maeyypa0JaeGymaedabaGaeyOhIukaniabggHiLdGccaWLjaGaaCzcamaabmaabaGaeGOmaiJaeGOmaidacaGLOaGaayzkaaaaaa@5878@

be the generating function for *M*_*t*_, *N*_*t*_. Muliplying both sides of (21) by *s*^*m *^*z*^*n *^and summing over *m *= l,... ∞; *n *= 0,...,∞ yields the partial differential equation

Ψ_*t *_= (*sz*^2^*λ *- (*λ *+ *μ*)*z *+ *μ*)Ψ_*z*_.     (23)

This equation was derived and solved by Kendall[7], using the method of characteristics. The solution is (for *λ *≠ *μ*)

Ψ(s,z;t)=β(sz−α)exp⁡(λαt)+α(β−sz)exp⁡(λβt)(sz−α)exp⁡(λαt)+(β−sz)exp⁡(λβt),     (24)
 MathType@MTEF@5@5@+=feaafiart1ev1aaatCvAUfKttLearuWrP9MDH5MBPbIqV92AaeXatLxBI9gBaebbnrfifHhDYfgasaacH8akY=wiFfYdH8Gipec8Eeeu0xXdbba9frFj0=OqFfea0dXdd9vqai=hGuQ8kuc9pgc9s8qqaq=dirpe0xb9q8qiLsFr0=vr0=vr0dc8meaabaqaciaacaGaaeqabaqabeGadaaakeaacqqHOoqwcqGGOaakcqWGZbWCcqGGSaalcqWG6bGEcqGG7aWocqWG0baDcqGGPaqkcqGH9aqpdaWcaaqaaGGaciab=j7aIjabcIcaOiabdohaZjabdQha6jabgkHiTiab=f7aHjabcMcaPiGbcwgaLjabcIha4jabcchaWjabcIcaOiab=T7aSjab=f7aHjabdsha0jabcMcaPiabgUcaRiab=f7aHjabcIcaOiab=j7aIjabgkHiTiabdohaZjabdQha6jabcMcaPiGbcwgaLjabcIha4jabcchaWjabcIcaOiab=T7aSjab=j7aIjabdsha0jabcMcaPaqaaiabcIcaOiabdohaZjabdQha6jabgkHiTiab=f7aHjabcMcaPiGbcwgaLjabcIha4jabcchaWjabcIcaOiab=T7aSjab=f7aHjabdsha0jabcMcaPiabgUcaRiabcIcaOiab=j7aIjabgkHiTiabdohaZjabdQha6jabcMcaPiGbcwgaLjabcIha4jabcchaWjabcIcaOiab=T7aSjab=j7aIjabdsha0jabcMcaPaaacqGGSaalcaWLjaGaaCzcamaabmaabaGaeGOmaiJaeGinaqdacaGLOaGaayzkaaaaaa@88F5@

where *α *= *α*(*s*), *β *= *β*(*s*) are the two (positive) roots of the quadratic equation

*λx*^2 ^- (*λ *+ *μ*)*x *+ *μs *= 0.     (25)

These roots are distinct for 0 ≤ *s *≤ 1, except when *λ *= *μ*, where the roots are distinct for 0 ≤ *s *≤ 1, but coincide for *s *= 1. We select *β*(*s*) to be the smaller root, so that

β(s)=12{1+μλ−(1+μλ)2−4μsλ}     (26)
 MathType@MTEF@5@5@+=feaafiart1ev1aaatCvAUfKttLearuWrP9MDH5MBPbIqV92AaeXatLxBI9gBaebbnrfifHhDYfgasaacH8akY=wiFfYdH8Gipec8Eeeu0xXdbba9frFj0=OqFfea0dXdd9vqai=hGuQ8kuc9pgc9s8qqaq=dirpe0xb9q8qiLsFr0=vr0=vr0dc8meaabaqaciaacaGaaeqabaqabeGadaaakeaaiiGacqWFYoGycqGGOaakcqWGZbWCcqGGPaqkcqGH9aqpdaWcaaqaaiabigdaXaqaaiabikdaYaaadaGadeqaaiabigdaXiabgUcaRmaalaaabaGae8hVd0gabaGae83UdWgaaiabgkHiTmaakaaabaWaaeWaaeaacqaIXaqmcqGHRaWkdaWcaaqaaiab=X7aTbqaaiab=T7aSbaaaiaawIcacaGLPaaadaahaaWcbeqaaiabikdaYaaakiabgkHiTmaalaaabaGaeGinaqJae8hVd0Maem4CamhabaGae83UdWgaaaWcbeaaaOGaay5Eaiaaw2haaiaaxMaacaWLjaWaaeWaaeaacqaIYaGmcqaI2aGnaiaawIcacaGLPaaaaaa@5062@

and note that *α*(1) = max{*λ*, *μ*}/*λ*, *β*(1) = min{*λ*, *μ*}/*λ *and *λ*[*α*(1) - *β*(1)] = |*λ *- *μ*|.

From (24) the individual generating function *ψ*_*M*_(*s*; *t*) = *E*(sMt
 MathType@MTEF@5@5@+=feaafiart1ev1aaatCvAUfKttLearuWrP9MDH5MBPbIqV92AaeXatLxBI9gBaebbnrfifHhDYfgasaacH8akY=wiFfYdH8Gipec8Eeeu0xXdbba9frFj0=OqFfea0dXdd9vqai=hGuQ8kuc9pgc9s8qqaq=dirpe0xb9q8qiLsFr0=vr0=vr0dc8meaabaqaciaacaGaaeqabaqabeGadaaakeaacqWGZbWCdaahaaWcbeqaaiabd2eannaaBaaameaacqWG0baDaeqaaaaaaaa@3109@) of *M*_*t *_(and similarly that of *N*_*t*_) can be derived. Specifically

ΨM(s;t)=E(sMt)=β(s−α)+α(β−s)e−λ(α−β)t(s−α)+(β−s)e−λ(α−β)t.     (27)
 MathType@MTEF@5@5@+=feaafiart1ev1aaatCvAUfKttLearuWrP9MDH5MBPbIqV92AaeXatLxBI9gBaebbnrfifHhDYfgasaacH8akY=wiFfYdH8Gipec8Eeeu0xXdbba9frFj0=OqFfea0dXdd9vqai=hGuQ8kuc9pgc9s8qqaq=dirpe0xb9q8qiLsFr0=vr0=vr0dc8meaabaqaciaacaGaaeqabaqabeGadaaakeaacqqHOoqwdaWgaaWcbaGaemyta0eabeaakiabcIcaOiabdohaZjabcUda7iabdsha0jabcMcaPiabg2da9iabdweafjabcIcaOiabdohaZnaaCaaaleqabaGaemyta00aaSbaaWqaaiabdsha0bqabaaaaOGaeiykaKIaeyypa0ZaaSaaaeaaiiGacqWFYoGycqGGOaakcqWGZbWCcqGHsislcqWFXoqycqGGPaqkcqGHRaWkcqWFXoqycqGGOaakcqWFYoGycqGHsislcqWGZbWCcqGGPaqkcqWGLbqzdaahaaWcbeqaaiabgkHiTiab=T7aSjabcIcaOiab=f7aHjabgkHiTiab=j7aIjabcMcaPiabdsha0baaaOqaaiabcIcaOiabdohaZjabgkHiTiab=f7aHjabcMcaPiabgUcaRiabcIcaOiab=j7aIjabgkHiTiabdohaZjabcMcaPiabdwgaLnaaCaaaleqabaGaeyOeI0Iae83UdWMaeiikaGIae8xSdeMaeyOeI0Iae8NSdiMaeiykaKIaemiDaqhaaaaakiabc6caUiaaxMaacaWLjaWaaeWaaeaacqaIYaGmcqaI3aWnaiaawIcacaGLPaaaaaa@768D@

Expanding this in a power-series expansion will yield the size distribution of the number of species which have existed by a finite time *t*. Simple closed-form expressions are not obtainable, but the expansion can be done numerically for specified parameter values using a computer mathematics program such as Maple VII[8]. It is easy to show that

E(Mt)=Ψ′M(1)=1+λλ−μ[e(λ−μ)t−1].     (28)
 MathType@MTEF@5@5@+=feaafiart1ev1aaatCvAUfKttLearuWrP9MDH5MBPbIqV92AaeXatLxBI9gBaebbnrfifHhDYfgasaacH8akY=wiFfYdH8Gipec8Eeeu0xXdbba9frFj0=OqFfea0dXdd9vqai=hGuQ8kuc9pgc9s8qqaq=dirpe0xb9q8qiLsFr0=vr0=vr0dc8meaabaqaciaacaGaaeqabaqabeGadaaakeaacqWGfbqrcqGGOaakcqWGnbqtdaWgaaWcbaGaemiDaqhabeaakiabcMcaPiabg2da9iqbfI6azzaafaWaaSbaaSqaaiabd2eanbqabaGccqGGOaakcqaIXaqmcqGGPaqkcqGH9aqpcqaIXaqmcqGHRaWkdaWcaaqaaGGaciab=T7aSbqaaiab=T7aSjabgkHiTiab=X7aTbaadaWadaqaaiabdwgaLnaaCaaaleqabaGaeiikaGIae83UdWMaeyOeI0Iae8hVd0MaeiykaKIaemiDaqhaaOGaeyOeI0IaeGymaedacaGLBbGaayzxaaGaeiOla4IaaCzcaiaaxMaadaqadaqaaiabikdaYiabiIda4aGaayjkaiaawMcaaaaa@5431@

Note that for *λ *> *μ*, *E*(*M*_*t*_) → ∞ as *t *→ ∞; while for *λ *<*μ*, *E*(*M*_*t*_) → *μ*/(*μ *- *λ*).

To find the distribution of the size of an extinct genus we consider the distribution of *M*_*t *_conditional on *N*(*t*) = 0. This has generating function Ω(*s*; *t*) = *E*(sMt
 MathType@MTEF@5@5@+=feaafiart1ev1aaatCvAUfKttLearuWrP9MDH5MBPbIqV92AaeXatLxBI9gBaebbnrfifHhDYfgasaacH8akY=wiFfYdH8Gipec8Eeeu0xXdbba9frFj0=OqFfea0dXdd9vqai=hGuQ8kuc9pgc9s8qqaq=dirpe0xb9q8qiLsFr0=vr0=vr0dc8meaabaqaciaacaGaaeqabaqabeGadaaakeaacqWGZbWCdaahaaWcbeqaaiabd2eannaaBaaameaacqWG0baDaeqaaaaaaaa@3109@|*N*_*t *_= 0) given by

Ω(s;t)=∑m=1∞pr(Mt=m|Nt=0)sm=Ψ(s,0;t)pr(Nt=0).     (29)
 MathType@MTEF@5@5@+=feaafiart1ev1aaatCvAUfKttLearuWrP9MDH5MBPbIqV92AaeXatLxBI9gBaebbnrfifHhDYfgasaacH8akY=wiFfYdH8Gipec8Eeeu0xXdbba9frFj0=OqFfea0dXdd9vqai=hGuQ8kuc9pgc9s8qqaq=dirpe0xb9q8qiLsFr0=vr0=vr0dc8meaabaqaciaacaGaaeqabaqabeGadaaakeaacqqHPoWvcqGGOaakcqWGZbWCcqGG7aWocqWG0baDcqGGPaqkcqGH9aqpdaaeWbqaaGqaaiab=bhaWjab=jhaYjabcIcaOiabd2eannaaBaaaleaacqWG0baDaeqaaOGaeyypa0JaemyBa0MaeiiFaWNaemOta40aaSbaaSqaaiabdsha0bqabaGccqGH9aqpcqaIWaamcqGGPaqkcqWGZbWCdaahaaWcbeqaaiabd2gaTbaaaeaacqWGTbqBcqGH9aqpcqaIXaqmaeaacqGHEisPa0GaeyyeIuoakiabg2da9maalaaabaGaeuiQdKLaeiikaGIaem4CamNaeiilaWIaeGimaaJaei4oaSJaemiDaqNaeiykaKcabaGae8hCaaNae8NCaiNaeiikaGIaemOta40aaSbaaSqaaiabdsha0bqabaGccqGH9aqpcqaIWaamcqGGPaqkaaGaeiOla4IaaCzcaiaaxMaadaqadaqaaiabikdaYiabiMda5aGaayjkaiaawMcaaaaa@67BC@

The probabilty of extinction by time *t *in the denominator can be evaluated as Ψ (1, 0; *t*) (or from standard results on birth and death processes) yielding

Ω(s;t)=[αβ(1−e−λ(α−β)t)α−βe−λ(α−β)t][max⁡{λ,μ}−min⁡{λ,μ}e−|λ−μ|tμ(1−e−|λ−μ|t)],     (30)
 MathType@MTEF@5@5@+=feaafiart1ev1aaatCvAUfKttLearuWrP9MDH5MBPbIqV92AaeXatLxBI9gBaebbnrfifHhDYfgasaacH8akY=wiFfYdH8Gipec8Eeeu0xXdbba9frFj0=OqFfea0dXdd9vqai=hGuQ8kuc9pgc9s8qqaq=dirpe0xb9q8qiLsFr0=vr0=vr0dc8meaabaqaciaacaGaaeqabaqabeGadaaakeaacqqHPoWvcqGGOaakcqWGZbWCcqGG7aWocqWG0baDcqGGPaqkcqGH9aqpdaWadaqaamaalaaabaacciGae8xSdeMae8NSdiMaeiikaGIaeGymaeJaeyOeI0Iaemyzau2aaWbaaSqabeaacqGHsislcqWF7oaBcqGGOaakcqWFXoqycqGHsislcqWFYoGycqGGPaqkcqWG0baDaaGccqGGPaqkaeaacqWFXoqycqWFsislcqWFYoGycqWGLbqzdaahaaWcbeqaaiabgkHiTiab=T7aSjabcIcaOiab=f7aHjabgkHiTiab=j7aIjabcMcaPiabdsha0baaaaaakiaawUfacaGLDbaadaWadaqaamaalaaabaGagiyBa0MaeiyyaeMaeiiEaGNaei4EaSNae83UdWMaeiilaWIae8hVd0MaeiyFa0NaeyOeI0IagiyBa0MaeiyAaKMaeiOBa4Maei4EaSNae83UdWMaeiilaWIae8hVd0MaeiyFa0Naemyzau2aaWbaaSqabeaacqGHsislcqGG8baFcqWF7oaBcqGHsislcqWF8oqBcqGG8baFcqWG0baDaaaakeaacqWF8oqBcqGGOaakcqaIXaqmcqGHsislcqWGLbqzdaahaaWcbeqaaiabgkHiTiabcYha8jab=T7aSjabgkHiTiab=X7aTjabcYha8jabdsha0baakiabcMcaPaaaaiaawUfacaGLDbaacqGGSaalcaWLjaGaaCzcamaabmaabaGaeG4mamJaeGimaadacaGLOaGaayzkaaaaaa@9387@

for *λ *≠ *μ*, and

Ω(s;t)=[αβ(1−e−λ(α−β)t)α−βe−λ(α−β)t][λt+1λt]     (31)
 MathType@MTEF@5@5@+=feaafiart1ev1aaatCvAUfKttLearuWrP9MDH5MBPbIqV92AaeXatLxBI9gBaebbnrfifHhDYfgasaacH8akY=wiFfYdH8Gipec8Eeeu0xXdbba9frFj0=OqFfea0dXdd9vqai=hGuQ8kuc9pgc9s8qqaq=dirpe0xb9q8qiLsFr0=vr0=vr0dc8meaabaqaciaacaGaaeqabaqabeGadaaakeaacqqHPoWvcqGGOaakcqWGZbWCcqGG7aWocqWG0baDcqGGPaqkcqGH9aqpdaWadaqaamaalaaabaacciGae8xSdeMae8NSdiMaeiikaGIaeGymaeJaeyOeI0Iaemyzau2aaWbaaSqabeaacqGHsislcqWF7oaBcqGGOaakcqWFXoqycqGHsislcqWFYoGycqGGPaqkcqWG0baDaaGccqGGPaqkaeaacqWFXoqycqWFsislcqWFYoGycqWGLbqzdaahaaWcbeqaaiabgkHiTiab=T7aSjabcIcaOiab=f7aHjabgkHiTiab=j7aIjabcMcaPiabdsha0baaaaaakiaawUfacaGLDbaadaWadaqaamaalaaabaGae83UdWMaemiDaqNaey4kaSIaeGymaedabaGae83UdWMaemiDaqhaaaGaay5waiaaw2faaiaaxMaacaWLjaWaaeWaaeaacqaIZaWmcqaIXaqmaiaawIcacaGLPaaaaaa@675C@

when *λ *= *μ*.

Since once a genus is extinct it remains extinct forever, the size distribution

qm†def¯¯Pr⁡{M∞=m|N∞=0}     (32)
MathType@MTEF@5@5@+=feaafiart1ev1aaatCvAUfKttLearuWrP9MDH5MBPbIqV92AaeXatLxBI9gBaebbnrfifHhDYfgasaacH8akY=wiFfYdH8Gipec8Eeeu0xXdbba9frFj0=OqFfea0dXdd9vqai=hGuQ8kuc9pgc9s8qqaq=dirpe0xb9q8qiLsFr0=vr0=vr0dc8meaabaqaciaacaGaaeqabaqabeGadaaakeaacqWGXbqCdaqhaaWcbaGaemyBa0gabaGaeiiiGyiaaOqbaeqabiqaaaqaamaameaabaGaeeizaqMaeeyzauMaeeOzaygaaaqaaaaacyGGqbaucqGGYbGCcqGG7bWEcqWGnbqtdaWgaaWcbaGaeyOhIukabeaakiabg2da9iabd2gaTjabcYha8jabd6eaonaaBaaaleaacqGHEisPaeqaaOGaeyypa0JaeGimaaJaeiyFa0NaaCzcaiaaxMaadaqadaqaaiabiodaZiabikdaYaGaayjkaiaawMcaaaaa@4ACC@

of an extinct fossil genus can be found by letting *t *→ ∞ in the generating function Ω(*s*; *t*) above. Since *α*(*s*) ≥ *β*(*s*), with the inequality strict for 0 ≤ *s *< 1, we have *e*^-*λ*(*α*-*β*)*t *^→ 0 as *t *→ ∞. Thus if we let *t *→ ∞ in the generating function above, we deduce that for all *λ *> 0 and *μ *> 0,

Ω(s;∞)=∑m=1∞qm†sm=max⁡{λ,μ}β(s)μ     (33)=(λ+μ)2min⁡{λ,μ}{1−1−4λμs(λ+μ)2}.(34)
 MathType@MTEF@5@5@+=feaafiart1ev1aaatCvAUfKttLearuWrP9MDH5MBPbIqV92AaeXatLxBI9gBaebbnrfifHhDYfgasaacH8akY=wiFfYdH8Gipec8Eeeu0xXdbba9frFj0=OqFfea0dXdd9vqai=hGuQ8kuc9pgc9s8qqaq=dirpe0xb9q8qiLsFr0=vr0=vr0dc8meaabaqaciaacaGaaeqabaqabeGadaaakeaafaqaaeGaeaaaaeaacqqHPoWvcqGGOaakcqWGZbWCcqGG7aWocqGHEisPcqGGPaqkaeaacqGH9aqpaeaadaaeWbqaaiabdghaXnaaDaaaleaacqWGTbqBaeaacqGGGaIHaaGccqWGZbWCdaahaaWcbeqaaiabd2gaTbaaaeaacqWGTbqBcqGH9aqpcqaIXaqmaeaacqGHEisPa0GaeyyeIuoakiabg2da9maalaaabaGagiyBa0MaeiyyaeMaeiiEaGNaei4EaShcciGae83UdWMaeiilaWIae8hVd0MaeiyFa0Nae8NSdiMaeiikaGIaem4CamNaeiykaKcabaGae8hVd0gaaiaaxMaacaWLjaaabaWaaeWaaeaacqaIZaWmcqaIZaWmaiaawIcacaGLPaaaaeaaaeaacqGH9aqpaeaadaWcaaqaaiabcIcaOiab=T7aSjabgUcaRiab=X7aTjabcMcaPaqaaiabikdaYiGbc2gaTjabcMgaPjabc6gaUjabcUha7jab=T7aSjabcYcaSiab=X7aTjabc2ha9baadaGadeqaaiabigdaXiabgkHiTmaakaaabaGaeGymaeJaeyOeI0YaaSaaaeaacqaI0aancqWF7oaBcqWF8oqBcqWGZbWCaeaacqGGOaakcqWF7oaBcqGHRaWkcqWF8oqBcqGGPaqkdaahaaWcbeqaaiabikdaYaaaaaaabeaaaOGaay5Eaiaaw2haaiabc6caUaqaamaabmaabaGaeG4mamJaeGinaqdacaGLOaGaayzkaaaaaaaa@8590@

Using the binomial theorem to expand the square root in (34) yields the pmf qm†
 MathType@MTEF@5@5@+=feaafiart1ev1aaatCvAUfKttLearuWrP9MDH5MBPbIqV92AaeXatLxBI9gBaebbnrfifHhDYfgasaacH8akY=wiFfYdH8Gipec8Eeeu0xXdbba9frFj0=OqFfea0dXdd9vqai=hGuQ8kuc9pgc9s8qqaq=dirpe0xb9q8qiLsFr0=vr0=vr0dc8meaabaqaciaacaGaaeqabaqabeGadaaakeaacqWGXbqCdaqhaaWcbaGaemyBa0gabaGaeiiiGyiaaaaa@30F5@ for the size of an extinct fossil genus. Where *m *≥ *n*_0 _= 1,

qm†=(λ+μ)min⁡{λ,μ}(2m−2)!(m−1)!m!(λμ)m(λ+μ)2m     (35)~(λ+μ)4π1/2min⁡{λ,μ}m3/2[4λμ(λ+μ)2]m.(36)
 MathType@MTEF@5@5@+=feaafiart1ev1aaatCvAUfKttLearuWrP9MDH5MBPbIqV92AaeXatLxBI9gBaebbnrfifHhDYfgasaacH8akY=wiFfYdH8Gipec8Eeeu0xXdbba9frFj0=OqFfea0dXdd9vqai=hGuQ8kuc9pgc9s8qqaq=dirpe0xb9q8qiLsFr0=vr0=vr0dc8meaabaqaciaacaGaaeqabaqabeGadaaakeaafaqaaeGaeaaaaeaacqWGXbqCdaqhaaWcbaGaemyBa0gabaGaeiiiGyiaaaGcbaGaeyypa0dabaWaaSaaaeaacqGGOaakiiGacqWF7oaBcqGHRaWkcqWF8oqBcqGGPaqkaeaacyGGTbqBcqGGPbqAcqGGUbGBcqGG7bWEcqWF7oaBcqGGSaalcqWF8oqBcqGG9bqFaaWaaSaaaeaacqGGOaakcqaIYaGmcqWGTbqBcqGHsislcqaIYaGmcqGGPaqkcqGGHaqiaeaacqGGOaakcqWGTbqBcqGHsislcqaIXaqmcqGGPaqkcqGGHaqicqWGTbqBcqGGHaqiaaWaaSaaaeaacqGGOaakcqaH7oaBcqWF8oqBcqGGPaqkdaahaaWcbeqaaiabd2gaTbaaaOqaaiabcIcaOiab=T7aSjabgUcaRiab=X7aTjabcMcaPmaaCaaaleqabaGaeGOmaiJaemyBa0gaaaaakiaaxMaacaWLjaaabaWaaeWaaeaacqaIZaWmcqaI1aqnaiaawIcacaGLPaaaaeaaaeaacqGG+bGFaeaadaWcaaqaaiabcIcaOiab=T7aSjabgUcaRiab=X7aTjabcMcaPaqaaiabisda0iab=b8aWnaaCaaaleqabaGaeGymaeJaei4la8IaeGOmaidaaOGagiyBa0MaeiyAaKMaeiOBa4Maei4EaSNae83UdWMaeiilaWIae8hVd0MaeiyFa0NaemyBa02aaWbaaSqabeaacqaIZaWmcqGGVaWlcqaIYaGmaaaaaOWaamWaaeaadaWcaaqaaiabisda0iab=T7aSjab=X7aTbqaaiabcIcaOiab=T7aSjabgUcaRiab=X7aTjabcMcaPmaaCaaaleqabaGaeGOmaidaaaaaaOGaay5waiaaw2faamaaCaaaleqabaGaemyBa0gaaOGaeiOla4cabaWaaeWaaeaacqaIZaWmcqaI2aGnaiaawIcacaGLPaaaaaaaaa@9696@

We observe that asymptotically *q*_*m *_decays faster than a power-law, except in the case when *λ *= *μ *when it follows a power law with exponent -3/2.

The expected size of an extinct genus can be found by evaluating the derivative Ω_*s*_(1; ∞), yielding

E(M∞|N∞=0)={λ/(λ−μ),λ>μ;∞λ=μ;μ/(μ−λ),λ<μ.     (37)
 MathType@MTEF@5@5@+=feaafiart1ev1aaatCvAUfKttLearuWrP9MDH5MBPbIqV92AaeXatLxBI9gBaebbnrfifHhDYfgasaacH8akY=wiFfYdH8Gipec8Eeeu0xXdbba9frFj0=OqFfea0dXdd9vqai=hGuQ8kuc9pgc9s8qqaq=dirpe0xb9q8qiLsFr0=vr0=vr0dc8meaabaqaciaacaGaaeqabaqabeGadaaakeaacqWGfbqrcqGGOaakcqWGnbqtdaWgaaWcbaGaeyOhIukabeaakiabcYha8jabd6eaonaaBaaaleaacqGHEisPaeqaaOGaeyypa0JaeGimaaJaeiykaKIaeyypa0ZaaiqabeaafaqaaeWacaaabaacciGae83UdWMaei4la8IaeiikaGIae83UdWMaeyOeI0Iae8hVd0MaeiykaKIaeiilaWcabaGae83UdWMaeyOpa4Jae8hVd0Maei4oaSdabaGaeyOhIukabaGae83UdWMaeyypa0Jae8hVd0Maei4oaSdabaGae8hVd0Maei4la8IaeiikaGIae8hVd0MaeyOeI0Iae83UdWMaeiykaKIaeiilaWcabaGae83UdWMaeyipaWJae8hVd0MaeiOla4caaaGaay5EaaGaaCzcaiaaxMaadaqadaqaaiabiodaZiabiEda3aGaayjkaiaawMcaaaaa@63E4@

The case *λ *= *μ *represents a phase transition analogous to the percolation phase transition (Hughes[9], Grimmett[10]). For this case although with probability one the genus goes extinct (*i.e*. *N*_∞ _= 0, w.p.1), the expected time for this to happen is infinite.

If there were initially *n*_0 _species in the genus, the expressions for the generating functions (24), (27) and (34) need to be modified by raising the expressions on the right-hand side to the *n*_0_th power. In particular, if we denote the pmf for the size of an extinct genus by qm†
 MathType@MTEF@5@5@+=feaafiart1ev1aaatCvAUfKttLearuWrP9MDH5MBPbIqV92AaeXatLxBI9gBaebbnrfifHhDYfgasaacH8akY=wiFfYdH8Gipec8Eeeu0xXdbba9frFj0=OqFfea0dXdd9vqai=hGuQ8kuc9pgc9s8qqaq=dirpe0xb9q8qiLsFr0=vr0=vr0dc8meaabaqaciaacaGaaeqabaqabeGadaaakeaacqWGXbqCdaqhaaWcbaGaemyBa0gabaGaeiiiGyiaaaaa@30F5@(*n*_0_) we have

∑m=n0∞qm†(n0)sn={(λ+μ)2min⁡{λ,μ}[1−1−4λμs(λ+μ)2]}n0.     (38)
 MathType@MTEF@5@5@+=feaafiart1ev1aaatCvAUfKttLearuWrP9MDH5MBPbIqV92AaeXatLxBI9gBaebbnrfifHhDYfgasaacH8akY=wiFfYdH8Gipec8Eeeu0xXdbba9frFj0=OqFfea0dXdd9vqai=hGuQ8kuc9pgc9s8qqaq=dirpe0xb9q8qiLsFr0=vr0=vr0dc8meaabaqaciaacaGaaeqabaqabeGadaaakeaadaaeWbqaaiabdghaXnaaDaaaleaacqWGTbqBaeaacqGGGaIHaaaabaGaemyBa0Maeyypa0JaemOBa42aaSbaaWqaaiabicdaWaqabaaaleaacqGHEisPa0GaeyyeIuoakiabcIcaOiabd6gaUnaaBaaaleaacqaIWaamaeqaaOGaeiykaKIaem4Cam3aaWbaaSqabeaacqWGUbGBaaGccqGH9aqpdaGadeqaamaalaaabaGaeiikaGccciGae83UdWMaey4kaSIae8hVd0MaeiykaKcabaGaeGOmaiJagiyBa0MaeiyAaKMaeiOBa4Maei4EaSNae83UdWMaeiilaWIae8hVd0MaeiyFa0haamaadmaabaGaeGymaeJaeyOeI0YaaOaaaeaacqaIXaqmcqGHsisldaWcaaqaaiabisda0iab=T7aSjab=X7aTjabdohaZbqaaiabcIcaOiab=T7aSjabgUcaRiab=X7aTjabcMcaPmaaCaaaleqabaGaeGOmaidaaaaaaeqaaaGccaGLBbGaayzxaaaacaGL7bGaayzFaaWaaWbaaSqabeaacqWGUbGBdaWgaaadbaGaeGimaadabeaaaaGccqGGUaGlcaWLjaGaaCzcamaabmaabaGaeG4mamJaeGioaGdacaGLOaGaayzkaaaaaa@7185@

We deduce at once from Eq. (38) that

qm†(n0)=[(λ+μ)2min⁡{λ,μ}]n0[4λμ(λ+μ)2]mQm(n0) (m≥n0),     (39)
 MathType@MTEF@5@5@+=feaafiart1ev1aaatCvAUfKttLearuWrP9MDH5MBPbIqV92AaeXatLxBI9gBaebbnrfifHhDYfgasaacH8akY=wiFfYdH8Gipec8Eeeu0xXdbba9frFj0=OqFfea0dXdd9vqai=hGuQ8kuc9pgc9s8qqaq=dirpe0xb9q8qiLsFr0=vr0=vr0dc8meaabaqaciaacaGaaeqabaqabeGadaaakeaafaqabeqacaaabaGaemyCae3aa0baaSqaaiabd2gaTbqaaiabcccigcaakiabcIcaOiabd6gaUnaaBaaaleaacqaIWaamaeqaaOGaeiykaKIaeyypa0ZaamWaaeaadaWcaaqaaiabcIcaOGGaciab=T7aSjabgUcaRiab=X7aTjabcMcaPaqaaiabikdaYiGbc2gaTjabcMgaPjabc6gaUjabcUha7jab=T7aSjabcYcaSiab=X7aTjabc2ha9baaaiaawUfacaGLDbaadaahaaWcbeqaaiabd6gaUnaaBaaameaacqaIWaamaeqaaaaakmaadmaabaWaaSaaaeaacqaI0aancqWF7oaBcqWF8oqBaeaacqGGOaakcqWF7oaBcqGHRaWkcqWF8oqBcqGGPaqkdaahaaWcbeqaaiabikdaYaaaaaaakiaawUfacaGLDbaadaahaaWcbeqaaiabd2gaTbaakiabdgfarnaaBaaaleaacqWGTbqBaeqaaOGaeiikaGIaemOBa42aaSbaaSqaaiabicdaWaqabaGccqGGPaqkaeaacqqGGaaicqGGOaakcqWGTbqBcqGHLjYScqWGUbGBdaWgaaWcbaGaeGimaadabeaakiabcMcaPiabcYcaSaaacaWLjaGaaCzcamaabmaabaGaeG4mamJaeGyoaKdacaGLOaGaayzkaaaaaa@7134@

where

∑m=n0∞Qm(n0)zm=[1−(1−z)1/2]n0.     (40)
 MathType@MTEF@5@5@+=feaafiart1ev1aaatCvAUfKttLearuWrP9MDH5MBPbIqV92AaeXatLxBI9gBaebbnrfifHhDYfgasaacH8akY=wiFfYdH8Gipec8Eeeu0xXdbba9frFj0=OqFfea0dXdd9vqai=hGuQ8kuc9pgc9s8qqaq=dirpe0xb9q8qiLsFr0=vr0=vr0dc8meaabaqaciaacaGaaeqabaqabeGadaaakeaadaaeWbqaaiabdgfarnaaBaaaleaacqWGTbqBaeqaaaqaaiabd2gaTjabg2da9iabd6gaUnaaBaaameaacqaIWaamaeqaaaWcbaGaeyOhIukaniabggHiLdGccqGGOaakcqWGUbGBdaWgaaWcbaGaeGimaadabeaakiabcMcaPiabdQha6naaCaaaleqabaGaemyBa0gaaOGaeyypa0Jaei4waSLaeGymaeJaeyOeI0IaeiikaGIaeGymaeJaeyOeI0IaemOEaONaeiykaKYaaWbaaSqabeaacqaIXaqmcqGGVaWlcqaIYaGmaaGccqGGDbqxdaahaaWcbeqaaiabd6gaUnaaBaaameaacqaIWaamaeqaaaaakiabc6caUiaaxMaacaWLjaWaaeWaaeaacqaI0aancqaIWaamaiaawIcacaGLPaaaaaa@5518@

The extraction of numerical values for the coefficients *Q*_*m*_(*n*_0_) for a modest fixed value of *n*_0 _is not difficult in practice. Alternatively, *Q*_*m*_(*n*_0_) can be found by a contour integral argument that we shall not write out here, leading to the formula

Qm(n0)=1π∑j=1︸j oddn0(n0j)sin⁡(jπ/2)Γ(j/2+1)Γ(m−j/2)Γ(m+1) (m≥n0).     (41)
 MathType@MTEF@5@5@+=feaafiart1ev1aaatCvAUfKttLearuWrP9MDH5MBPbIqV92AaeXatLxBI9gBaebbnrfifHhDYfgasaacH8akY=wiFfYdH8Gipec8Eeeu0xXdbba9frFj0=OqFfea0dXdd9vqai=hGuQ8kuc9pgc9s8qqaq=dirpe0xb9q8qiLsFr0=vr0=vr0dc8meaabaqaciaacaGaaeqabaqabeGadaaakeaacqWGrbqudaWgaaWcbaGaemyBa0gabeaakiabcIcaOiabd6gaUnaaBaaaleaacqaIWaamaeqaaOGaeiykaKIaeyypa0ZaaSaaaeaacqaIXaqmaeaaiiGacqWFapaCaaWaaabCaeaadaqadaqaauaabeqaceaaaeaacqWGUbGBdaWgaaWcbaGaeGimaadabeaaaOqaaiabdQgaQbaaaiaawIcacaGLPaaaaSqaamaayaaabaGaemOAaOMaeyypa0JaeGymaedameaacqWGQbGAcqqGGaaiieaacqGFVbWBcqGFKbazcqGFKbazaSGaayjo+daabaGaemOBa42aaSbaaWqaaiabicdaWaqabaaaniabggHiLdGccyGGZbWCcqGGPbqAcqGGUbGBcqGGOaakcqWGQbGAcqWFapaCcqGGVaWlcqaIYaGmcqGGPaqkdaWcaaqaaiabfo5ahjabcIcaOiabdQgaQjabc+caViabikdaYiabgUcaRiabigdaXiabcMcaPiabfo5ahjabcIcaOiabd2gaTjabgkHiTiabdQgaQjabc+caViabikdaYiabcMcaPaqaaiabfo5ahjabcIcaOiabd2gaTjabgUcaRiabigdaXiabcMcaPaaacqqGGaaicqGGOaakcqWGTbqBcqGHLjYScqWGUbGBdaWgaaWcbaGaeGimaadabeaakiabcMcaPiabc6caUiaaxMaacaWLjaWaaeWaaeaacqaI0aancqaIXaqmaiaawIcacaGLPaaaaaa@7CE7@

In particular, the following simple formula holds for *n*_0 _= 1, 2, 3 or 4:

Qm(n0)=n0(2m−2)!22m−1(m−1)!m!{1−(n0−1)(n0−2)4(m−3/2)},m≥n0.
 MathType@MTEF@5@5@+=feaafiart1ev1aaatCvAUfKttLearuWrP9MDH5MBPbIqV92AaeXatLxBI9gBaebbnrfifHhDYfgasaacH8akY=wiFfYdH8Gipec8Eeeu0xXdbba9frFj0=OqFfea0dXdd9vqai=hGuQ8kuc9pgc9s8qqaq=dirpe0xb9q8qiLsFr0=vr0=vr0dc8meaabaqaciaacaGaaeqabaqabeGadaaakeaafaqabeqacaaabaGaemyuae1aaSbaaSqaaiabd2gaTbqabaGccqGGOaakcqWGUbGBdaWgaaWcbaGaeGimaadabeaakiabcMcaPiabg2da9maalaaabaGaemOBa42aaSbaaSqaaiabicdaWaqabaGccqGGOaakcqaIYaGmcqWGTbqBcqGHsislcqaIYaGmcqGGPaqkcqGGHaqiaeaacqaIYaGmdaahaaWcbeqaaiabikdaYiabd2gaTjabgkHiTiabigdaXaaakiabcIcaOiabd2gaTjabgkHiTiabigdaXiabcMcaPiabcgcaHiabd2gaTjabcgcaHaaacqGG7bWEcqaIXaqmcqGHsisldaWcaaqaaiabcIcaOiabd6gaUnaaBaaaleaacqaIWaamaeqaaOGaeyOeI0IaeGymaeJaeiykaKIaeiikaGIaemOBa42aaSbaaSqaaiabicdaWaqabaGccqGHsislcqaIYaGmcqGGPaqkaeaacqaI0aancqGGOaakcqWGTbqBcqGHsislcqaIZaWmcqGGVaWlcqaIYaGmcqGGPaqkaaGaeiyFa0NaeiilaWcabaGaemyBa0MaeyyzImRaemOBa42aaSbaaSqaaiabicdaWaqabaaaaOGaeiOla4caaa@6BA4@

From Eqs (39) and (41) we see that for arbitrary fixed *n*_0 _≥ 1,

qm†(n0)~[λ+μ2min⁡{λ,μ}]n0[4λμ(λ+μ)2]mn02π1/2m3/2
 MathType@MTEF@5@5@+=feaafiart1ev1aaatCvAUfKttLearuWrP9MDH5MBPbIqV92AaeXatLxBI9gBaebbnrfifHhDYfgasaacH8akY=wiFfYdH8Gipec8Eeeu0xXdbba9frFj0=OqFfea0dXdd9vqai=hGuQ8kuc9pgc9s8qqaq=dirpe0xb9q8qiLsFr0=vr0=vr0dc8meaabaqaciaacaGaaeqabaqabeGadaaakeaacqWGXbqCdaqhaaWcbaGaemyBa0gabaGaeiiiGyiaaOGaeiikaGIaemOBa42aaSbaaSqaaiabicdaWaqabaGccqGGPaqkcqGG+bGFdaWadaqaamaalaaabaacciGae83UdWMaey4kaSIae8hVd0gabaGaeGOmaiJagiyBa0MaeiyAaKMaeiOBa4Maei4EaSNae83UdWMaeiilaWIae8hVd0MaeiyFa0haaaGaay5waiaaw2faamaaCaaaleqabaGaemOBa42aaSbaaWqaaiabicdaWaqabaaaaOWaamWaaeaadaWcaaqaaiabisda0iab=T7aSjab=X7aTbqaaiabcIcaOiab=T7aSjabgUcaRiab=X7aTjabcMcaPmaaCaaaleqabaGaeGOmaidaaaaaaOGaay5waiaaw2faamaaCaaaleqabaGaemyBa0gaaOWaaSaaaeaacqWGUbGBdaWgaaWcbaGaeGimaadabeaaaOqaaiabikdaYiab=b8aWnaaCaaaleqabaGaeGymaeJaei4la8IaeGOmaidaaOGaemyBa02aaWbaaSqabeaacqaIZaWmcqGGVaWlcqaIYaGmaaaaaaaa@67C6@

as *m *→ ∞. The right-hand side of this differs from that of (36) only by a multiplicative constant, and for all *n*_0 _≥ 1 asymptotically qm†
 MathType@MTEF@5@5@+=feaafiart1ev1aaatCvAUfKttLearuWrP9MDH5MBPbIqV92AaeXatLxBI9gBaebbnrfifHhDYfgasaacH8akY=wiFfYdH8Gipec8Eeeu0xXdbba9frFj0=OqFfea0dXdd9vqai=hGuQ8kuc9pgc9s8qqaq=dirpe0xb9q8qiLsFr0=vr0=vr0dc8meaabaqaciaacaGaaeqabaqabeGadaaakeaacqWGXbqCdaqhaaWcbaGaemyBa0gabaGaeiiiGyiaaaaa@30F5@(*n*_0_) decays faster than a power law except in the case *λ *= *μ*, when it follows a power law with exponent -3/2.

**Figure 2:**
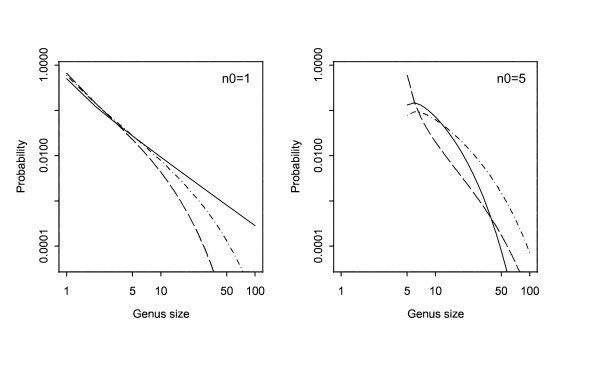
Logarithmic plots (both scales logarithmic) of the size distribution of genera, assuming only background extinctions. The left-hand plot is for *n*_0 _= 1 and the right-hand one for *n*_0 _= 5. For both plots *λ *= 1. For the sake of display the points of the probability mass function have been joined by lines:- solid (*μ *= 1); broken (*μ *= 1.5) and dot-dash (*μ *= 0.5).

Fig. 2 shows the distribution of the size of an extinct genus plotted on logarithmic axes, for two values of *n*_0 _and three values of *μ *with *λ *= 1. In the case *n*_0 _= 1 (left-hand panel), an approximate power-law distribution (straight-line plot) can be seen in the case of equal birth and death rates (*λ *= *μ*, the solid line). When the birth and death rates differ (*λ *≠ *μ*) there is departure from the power-law with faster decay in probabilities as genus size increases both when *λ *> *μ *and when *λ *<*μ*. In the case when the initial size *n*_0 _of the pioneering genus exceeds one (right-hand panel), similar results pertain asymptotically (large genus sizes), but perturbations in the size distribution occur at the lower end (around *n*_0_).

## Both background and cataclysmic extinctions

We have very limited results in the case. The difficulty lies in the fact that at the time (*τ*, say) at which the cataclysmic extinction event occurs, different genera will have been in existence for different lengths of time. Unlike the case discussed in an earlier (no background extinctions) where we established that the times of establishment of new genera formed an order-statistic process, whence it followed that at time *τ*, the times in existence of distinct genera constituted iid random variables with a truncated exponential distribution, in the present case (with background extinctions) we have not been able to establish that the times of establishment of new genera constitute an order-statistic process. Thus it has not been possible to determine the size distribution of derived genera, destroyed in the cataclysm, since their time in existence is unknown. This is particularly unfortunate, since it seems that in fact for many fossil families both background and cataclysmic extinctions have occurred (Raup and Sepkoski [11]).

The only genus for which the time in existence is known is the pioneering genus. The pgf of the size of this genus is given by [ΦM(s;τ)]n0
 MathType@MTEF@5@5@+=feaafiart1ev1aaatCvAUfKttLearuWrP9MDH5MBPbIqV92AaeXatLxBI9gBaebbnrfifHhDYfgasaacH8akY=wiFfYdH8Gipec8Eeeu0xXdbba9frFj0=OqFfea0dXdd9vqai=hGuQ8kuc9pgc9s8qqaq=dirpe0xb9q8qiLsFr0=vr0=vr0dc8meaabaqaciaacaGaaeqabaqabeGadaaakeaadaWadaqaaiabfA6agnaaBaaaleaacqWGnbqtaeqaaOWaaeWaaeaacqWGZbWCcqGG7aWoiiGacqWFepaDaiaawIcacaGLPaaaaiaawUfacaGLDbaadaahaaWcbeqaaiabd6gaUnaaBaaameaacqaIWaamaeqaaaaaaaa@39E0@ where Φ_*M *_is defined in (27). This cannot be expanded in terms of simple functions to obtain explicit probabilities for sizes, although of course it can always be done numerically for specific parameter values. The expected size of the pioneering genus is

E(Mpion)=n0(1+λλ−μ[e(λ−μ)τ−1]).     (42)
 MathType@MTEF@5@5@+=feaafiart1ev1aaatCvAUfKttLearuWrP9MDH5MBPbIqV92AaeXatLxBI9gBaebbnrfifHhDYfgasaacH8akY=wiFfYdH8Gipec8Eeeu0xXdbba9frFj0=OqFfea0dXdd9vqai=hGuQ8kuc9pgc9s8qqaq=dirpe0xb9q8qiLsFr0=vr0=vr0dc8meaabaqaciaacaGaaeqabaqabeGadaaakeaacqWGfbqrcqGGOaakcqWGnbqtdaWgaaWcbaGaemiCaaNaemyAaKMaem4Ba8MaemOBa4gabeaakiabcMcaPiabg2da9iabd6gaUnaaBaaaleaacqaIWaamaeqaaOWaaeWaaeaacqaIXaqmcqGHRaWkdaWcaaqaaGGaciab=T7aSbqaaiab=T7aSjabgkHiTiab=X7aTbaadaWadaqaaiabdwgaLnaaCaaaleqabaGaeiikaGIae83UdWMaeyOeI0Iae8hVd0MaeiykaKIae8hXdqhaaOGaeyOeI0IaeGymaedacaGLBbGaayzxaaaacaGLOaGaayzkaaGaeiOla4IaaCzcaiaaxMaadaqadaqaaiabisda0iabikdaYaGaayjkaiaawMcaaaaa@560D@

## 1 Size distribution of families

In this section we consider the number of *genera *in the *family *derived from the pioneering species, assuming (as in the second section) that new genera are created by extreme speciations (at probabilistic rate *γ*) and (as in the third section) that background extinctions occur at the rate *μ*.

It can be shown (see Appendix) that the number of genera, *G*_*t*_, which have existed up to time *t *has a generating function Φ_*G*_(*s*; *t*) = *E*(sGt
 MathType@MTEF@5@5@+=feaafiart1ev1aaatCvAUfKttLearuWrP9MDH5MBPbIqV92AaeXatLxBI9gBaebbnrfifHhDYfgasaacH8akY=wiFfYdH8Gipec8Eeeu0xXdbba9frFj0=OqFfea0dXdd9vqai=hGuQ8kuc9pgc9s8qqaq=dirpe0xb9q8qiLsFr0=vr0=vr0dc8meaabaqaciaacaGaaeqabaqabeGadaaakeaacqWGZbWCdaahaaWcbeqaaiabdEeahnaaBaaameaacqWG0baDaeqaaaaaaaa@30FD@) given by

ΦG(s;t)=s[(λ+γ)λ+γsΨ˜(λ+γsλ+γ;t)]n0,     (43)
 MathType@MTEF@5@5@+=feaafiart1ev1aaatCvAUfKttLearuWrP9MDH5MBPbIqV92AaeXatLxBI9gBaebbnrfifHhDYfgasaacH8akY=wiFfYdH8Gipec8Eeeu0xXdbba9frFj0=OqFfea0dXdd9vqai=hGuQ8kuc9pgc9s8qqaq=dirpe0xb9q8qiLsFr0=vr0=vr0dc8meaabaqaciaacaGaaeqabaqabeGadaaakeaacqqHMoGrdaWgaaWcbaGaem4raCeabeaakiabcIcaOiabdohaZjabcUda7iabdsha0jabcMcaPiabg2da9iabdohaZnaadmaabaWaaSaaaeaacqGGOaakiiGacqWF7oaBcqGHRaWkcqWFZoWzcqGGPaqkaeaacqWF7oaBcqGHRaWkcqWFZoWzcqWGZbWCaaGafuiQdKLbaGaadaqadaqaamaalaaabaGae83UdWMaey4kaSIae83SdCMaem4CamhabaGae83UdWMaey4kaSIae83SdCgaaiabcUda7iabdsha0bGaayjkaiaawMcaaaGaay5waiaaw2faamaaCaaaleqabaGaemOBa42aaSbaaWqaaiabicdaWaqabaaaaOGaeiilaWIaaCzcaiaaxMaadaqadaqaaiabisda0iabiodaZaGaayjkaiaawMcaaaaa@5CD6@

where Ψ˜
 MathType@MTEF@5@5@+=feaafiart1ev1aaatCvAUfKttLearuWrP9MDH5MBPbIqV92AaeXatLxBI9gBaebbnrfifHhDYfgasaacH8akY=wiFfYdH8Gipec8Eeeu0xXdbba9frFj0=OqFfea0dXdd9vqai=hGuQ8kuc9pgc9s8qqaq=dirpe0xb9q8qiLsFr0=vr0=vr0dc8meaabaqaciaacaGaaeqabaqabeGadaaakeaacuqHOoqwgaacaaaa@2E4A@ is the same as Ψ_*M *_in (27), but with *λ *replaced by *λ *+ *γ*. This can be verified directly in the case *μ *= 0 (only cataclysmic extinctions) for which *G*_*t *_≡ *K*_*t *_(see second section) with *G*_*t *_+ *n*_0 _- 1 having a negative binomial distribution. In the more general case the proof is somewhat technical and is relegated to the Appendix. The expected number of genera in the family can easily be determined from (43) as

E(Gt)=1+n0γλ+γ−μ(e(λ+γ−μ)t−1).     (44)
 MathType@MTEF@5@5@+=feaafiart1ev1aaatCvAUfKttLearuWrP9MDH5MBPbIqV92AaeXatLxBI9gBaebbnrfifHhDYfgasaacH8akY=wiFfYdH8Gipec8Eeeu0xXdbba9frFj0=OqFfea0dXdd9vqai=hGuQ8kuc9pgc9s8qqaq=dirpe0xb9q8qiLsFr0=vr0=vr0dc8meaabaqaciaacaGaaeqabaqabeGadaaakeaacqWGfbqrcqGGOaakcqWGhbWrdaWgaaWcbaGaemiDaqhabeaakiabcMcaPiabg2da9iabigdaXiabgUcaRiabd6gaUnaaBaaaleaacqaIWaamaeqaaOWaaSaaaeaaiiGacqWFZoWzaeaacqWF7oaBcqGHRaWkcqWFZoWzcqGHsislcqWF8oqBaaWaaeWaaeaacqWGLbqzdaahaaWcbeqaaiabcIcaOiab=T7aSjabgUcaRiab=n7aNjabgkHiTiab=X7aTjabcMcaPiabdsha0baakiabgkHiTiabigdaXaGaayjkaiaawMcaaiabc6caUiaaxMaacaWLjaWaaeWaaeaacqaI0aancqaI0aanaiaawIcacaGLPaaaaaa@54A0@

**Figure 3:**
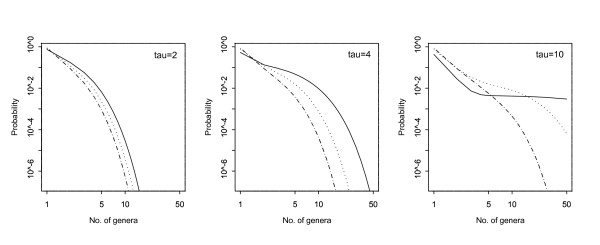
Logarithmic plots (both scales logarithmic) of the distribution of the number of genera in a family, assuming background and cataclysmic extinctions. The three panels (from left to right) correspond to *τ *= 2,4 and 10. In all cases *λ *= 1; *γ *= 0.1; *n*_0 _= 1. For the sake of display the points of the probability mass function have been joined by lines:- solid (*μ *= 1); dotted (*μ *= 1.5) and dot-dash (*μ *= 0.5).

If, following a cataclysmic event from which *n*_0 _species survived, a subsequent cataclysm occurred *τ *time units later, the size distribution of the family (number of genera) derived from these *n*_0 _pioneering species, would have pgf Φ_*G*_(*s*; *τ*). While no simple expansion of this is possible it can be done numerically. Some examples are shown in Fig. 3. The distributions show considerable deviation from a power law (straight line in logarithmic plots). They appear similar to the corresponding distributions of number of species in a genus (Fig. 1, top row) for smaller values of *τ*, but are further from the power-law form for larger *τ*. Thus it would appear that under the birth-and-death model power-law (fractal-like) size distributions are less likely to occur at higher taxonomic levels.

## Monotypic taxa

One characteristic of interest in the empirical study of lineages is the proportion of monotypic taxa. Przeworski and Wall[5] compared the proportions of monospecific genera and of monogeneric families observed in the fossil record with results from a simulation of a birth-and-death process model. In this section we compute probabilities of such monotypic taxa. We consider the cases of (1) only background extinctions; and (2) only cataclysmic extinctions.

### Only background extinctions

For a genus in existence for *t *time units, the probability of it having only ever contained one species by that time is

Pr⁡(Mt=1)=Ψ′M(0;t)=lim⁡s→0ΨM(s;t)s=λe−(λ+μ)t+μλ+μ     (45)
 MathType@MTEF@5@5@+=feaafiart1ev1aaatCvAUfKttLearuWrP9MDH5MBPbIqV92AaeXatLxBI9gBaebbnrfifHhDYfgasaacH8akY=wiFfYdH8Gipec8Eeeu0xXdbba9frFj0=OqFfea0dXdd9vqai=hGuQ8kuc9pgc9s8qqaq=dirpe0xb9q8qiLsFr0=vr0=vr0dc8meaabaqaciaacaGaaeqabaqabeGadaaakeaacyGGqbaucqGGYbGCcqGGOaakcqWGnbqtdaWgaaWcbaGaemiDaqhabeaakiabg2da9iabigdaXiabcMcaPiabg2da9iqbfI6azzaafaWaaSbaaSqaaiabd2eanbqabaGccqGGOaakcqaIWaamcqGG7aWocqWG0baDcqGGPaqkcqGH9aqpdaWfqaqaaiGbcYgaSjabcMgaPjabc2gaTbWcbaGaem4CamNaeyOKH4QaeGimaadabeaakmaalaaabaGaeuiQdK1aaSbaaSqaaiabd2eanbqabaGccqGGOaakcqWGZbWCcqGG7aWocqWG0baDcqGGPaqkaeaacqWGZbWCaaGaeyypa0ZaaSaaaeaaiiGacqWF7oaBcqWGLbqzdaahaaWcbeqaaiabgkHiTiabcIcaOiab=T7aSjabgUcaRiab=X7aTjabcMcaPiabdsha0baakiabgUcaRiab=X7aTbqaaiab=T7aSjabgUcaRiab=X7aTbaacaWLjaGaaCzcamaabmaabaGaeGinaqJaeGynaudacaGLOaGaayzkaaaaaa@6AA2@

where Ψ_*M *_is as in (27). Since all extinct fossil genera are finite in size, the probability of such a genus being monospecific is (from the results in fourth section)

Pr(monospecificgenus)=Pr(M∞=1|M∞<∞)={μλ+μ,λ≤μ,λλ+μ,λ>μ.     (46)
 MathType@MTEF@5@5@+=feaafiart1ev1aaatCvAUfKttLearuWrP9MDH5MBPbIqV92AaeXatLxBI9gBaebbnrfifHhDYfgasaacH8akY=wiFfYdH8Gipec8Eeeu0xXdbba9frFj0=OqFfea0dXdd9vqai=hGuQ8kuc9pgc9s8qqaq=dirpe0xb9q8qiLsFr0=vr0=vr0dc8meaabaqaciaacaGaaeqabaqabeGadaaakeaaieaacqWFqbaucqWFYbGCcqGGOaakcqWFTbqBcqWFVbWBcqWFUbGBcqWFVbWBcqWFZbWCcqWFWbaCcqWFLbqzcqWFJbWycqWFPbqAcqWFMbGzcqWFPbqAcqWFJbWycqWFGaaicqWFNbWzcqWFLbqzcqWFUbGBcqWF1bqDcqWFZbWCcqGGPaqkcqGH9aqpcqqGqbaucqWFYbGCcqGGOaakcqWGnbqtdaWgaaWcbaGaeyOhIukabeaakiabg2da9iabigdaXiabcYha8jabd2eannaaBaaaleaacqGHEisPaeqaaOGaeyipaWJaeyOhIuQaeiykaKIaeyypa0ZaaiqabeaafaqaaeGacaaabaWaaSaaaeaaiiGacqGF8oqBaeaacqGF7oaBcqGHRaWkcqGF8oqBaaGaeiilaWcabaGae43UdWMaeyizImQae4hVd0MaeiilaWcabaWaaSaaaeaacqGF7oaBaeaacqGF7oaBcqGHRaWkcqGF8oqBaaGaeiilaWcabaGae43UdWMaeyOpa4Jae4hVd0MaeiOla4caaaGaay5EaaGaaCzcaiaaxMaadaqadaqaaiabisda0iabiAda2aGaayjkaiaawMcaaaaa@798C@

Note that this is never less than one half (with this minimum value occurring when *λ *= *μ*), so in the absence of cataclysmic extinctions, one should expect at least half of all extinct genera to be monospecific.

Consider now the distribution of the number of *genera *derived from a pioneering genus with *n*_0 _species. Again since all observed extinct families will be of finite size, the probability of such a fossil family being monogeneric is

Pr⁡(G∞=1|G∞<∞)={(λ+γμ)n0Φ′G(0,∞),λ+γ>μ,Φ′G(0,∞),λ+γ≤μ.     (47)
 MathType@MTEF@5@5@+=feaafiart1ev1aaatCvAUfKttLearuWrP9MDH5MBPbIqV92AaeXatLxBI9gBaebbnrfifHhDYfgasaacH8akY=wiFfYdH8Gipec8Eeeu0xXdbba9frFj0=OqFfea0dXdd9vqai=hGuQ8kuc9pgc9s8qqaq=dirpe0xb9q8qiLsFr0=vr0=vr0dc8meaabaqaciaacaGaaeqabaqabeGadaaakeaacyGGqbaucqGGYbGCcqGGOaakcqWGhbWrdaWgaaWcbaGaeyOhIukabeaakiabg2da9iabigdaXiabcYha8jabdEeahnaaBaaaleaacqGHEisPaeqaaOGaeyipaWJaeyOhIuQaeiykaKIaeyypa0ZaaiqabeaafaqaaeGacaaabaWaaeWaaeaadaWcaaqaaGGaciab=T7aSjabgUcaRiab=n7aNbqaaiab=X7aTbaaaiaawIcacaGLPaaadaahaaWcbeqaaiabd6gaUnaaBaaameaacqaIWaamaeqaaaaakiqbfA6agzaafaWaaSbaaSqaaiabdEeahbqabaGccqGGOaakcqaIWaamcqGGSaalcqGHEisPcqGGPaqkcqGGSaalaeaacqWF7oaBcqGHRaWkcqWFZoWzcqGH+aGpcqWF8oqBcqGGSaalaeaacuqHMoGrgaqbamaaBaaaleaacqWGhbWraeqaaOGaeiikaGIaeGimaaJaeiilaWIaeyOhIuQaeiykaKIaeiilaWcabaGae83UdWMaey4kaSIae83SdCMaeyizImQae8hVd0MaeiOla4caaaGaay5EaaGaaCzcaiaaxMaadaqadaqaaiabisda0iabiEda3aGaayjkaiaawMcaaaaa@6F1B@

where

Φ′G(0,∞)=∂∂sΦG(s,∞)|s=0=[((λ+γ)λ)ΨL(λλ+γ;∞)]n0
 MathType@MTEF@5@5@+=feaafiart1ev1aaatCvAUfKttLearuWrP9MDH5MBPbIqV92AaeXatLxBI9gBaebbnrfifHhDYfgasaacH8akY=wiFfYdH8Gipec8Eeeu0xXdbba9frFj0=OqFfea0dXdd9vqai=hGuQ8kuc9pgc9s8qqaq=dirpe0xb9q8qiLsFr0=vr0=vr0dc8meaabaqaciaacaGaaeqabaqabeGadaaakeaacuqHMoGrgaqbamaaBaaaleaacqWGhbWraeqaaOGaeiikaGIaeGimaaJaeiilaWIaeyOhIuQaeiykaKIaeyypa0ZaaSaaaeaacqGHciITaeaacqGHciITcqWGZbWCaaGaeuOPdy0aaSbaaSqaaiabdEeahbqabaGccqGGOaakcqWGZbWCcqGGSaalcqGHEisPcqGGPaqkcqGG8baFdaWgaaWcbaGaem4CamNaeyypa0JaeGimaadabeaakiabg2da9maadmaabaWaaeWaaeaadaWcaaqaaiabcIcaOGGaciab=T7aSjabgUcaRiab=n7aNjabcMcaPaqaaiab=T7aSbaaaiaawIcacaGLPaaacqqHOoqwdaWgaaWcbaGaemitaWeabeaakmaabmaabaWaaSaaaeaacqWF7oaBaeaacqWF7oaBcqGHRaWkcqWFZoWzaaGaei4oaSJaeyOhIukacaGLOaGaayzkaaaacaGLBbGaayzxaaWaaWbaaSqabeaacqWGUbGBdaWgaaadbaGaeGimaadabeaaaaaaaa@62C1@

using (43). Thus, using (34), when *λ *+ *γ *> *μ*

Pr(monogeneric family)=[λ+γ2λμ(λ+γ+μ−(λ+γ+μ)2−4λμ)]n0;
 MathType@MTEF@5@5@+=feaafiart1ev1aaatCvAUfKttLearuWrP9MDH5MBPbIqV92AaeXatLxBI9gBaebbnrfifHhDYfgasaacH8akY=wiFfYdH8Gipec8Eeeu0xXdbba9frFj0=OqFfea0dXdd9vqai=hGuQ8kuc9pgc9s8qqaq=dirpe0xb9q8qiLsFr0=vr0=vr0dc8meaabaqaciaacaGaaeqabaqabeGadaaakeaaieaacqWFqbaucqWFYbGCcqGGOaakcqWFTbqBcqWFVbWBcqWFUbGBcqWFVbWBcqWFNbWzcqWFLbqzcqWFUbGBcqWFLbqzcqWFYbGCcqWFPbqAcqWFJbWycqqGGaaicqWFMbGzcqWFHbqycqWFTbqBcqWFPbqAcqWFSbaBcqWF5bqEcqGGPaqkcqGH9aqpdaWadaqaamaalaaabaacciGae43UdWMaey4kaSIae43SdCgabaGaeGOmaiJae43UdWMae4hVd0gaamaabmaabaGae43UdWMaey4kaSIae43SdCMaey4kaSIae4hVd0MaeyOeI0YaaOaaaeaacqGGOaakcqGF7oaBcqGHRaWkcqGFZoWzcqGHRaWkcqGF8oqBcqGGPaqkdaahaaWcbeqaaiabikdaYaaakiabgkHiTiabisda0iab+T7aSjab+X7aTbWcbeaaaOGaayjkaiaawMcaaaGaay5waiaaw2faamaaCaaaleqabaGaemOBa42aaSbaaWqaaiabicdaWaqabaaaaOGaei4oaSdaaa@7048@

and when *λ *+ *γ *≤ *μ*, the right hand side is modified by the fraction (*λ *+ *γ*)/(2*λμ*) being replaced by 1/(2*λ*).

Comparing the probability of a monospecific genus with that of a mono-generic family is complicated in general because of the number of parameters. But one can show that with *n*_0 _= 1, the probability of a monogeneric family always exceeds that of a monospecific genus if the rate of formation of new genera is suitably small - *i.e*. if 0 <*γ *<*γ*_0_, for some positive *γ*_0 _(depending on *λ *and *μ*). In this case of course the probability of a monogeneric family will also exceed 0.5.

### Only cataclysmic extinctions

If a cataclysmic extinction event occurs at time *τ*, the probabilities of a monotypic genus and of a monogeneric family can be found easily from the results of the second section using the explicit expressions for the generating functions of the number of species *N*_*τ*_, (8); and for the number of genera *L*_*τ*_, (6). Specifically if there is initially a single species in the genus the probability that it is monospecific at the time of extinction is

Pr(monospecific genus) = Pr(*N*_*τ *_= 1) = e^-*λτ*^,     (48)

which is simply the probabilty of no speciations in (0, *τ*). In contrast the probabilty of a monogeneric family is

Pr⁡(monogeneric family)=Pr(Kτ=1)=[p(τ)]n0=[(λ+γ)e−(λ+γ)τγ+λe−(λ+γ)τ]n0.     (49)
 MathType@MTEF@5@5@+=feaafiart1ev1aaatCvAUfKttLearuWrP9MDH5MBPbIqV92AaeXatLxBI9gBaebbnrfifHhDYfgasaacH8akY=wiFfYdH8Gipec8Eeeu0xXdbba9frFj0=OqFfea0dXdd9vqai=hGuQ8kuc9pgc9s8qqaq=dirpe0xb9q8qiLsFr0=vr0=vr0dc8meaabaqaciaacaGaaeqabaqabeGadaaakeaacyGGqbaucqGGYbGCcqGGOaakieaacqWFTbqBcqWFVbWBcqWFUbGBcqWFVbWBcqWFNbWzcqWFLbqzcqWFUbGBcqWFLbqzcqWFYbGCcqWFPbqAcqWFJbWycqqGGaaicqWFMbGzcqWFHbqycqWFTbqBcqWFPbqAcqWFSbaBcqWF5bqEcqGGPaqkcqGH9aqpcqWFqbaucqWFYbGCcqGGOaakcqWGlbWsdaWgaaWcbaacciGae4hXdqhabeaakiabg2da9iabigdaXiabcMcaPiabg2da9iabcUfaBjabdchaWjabcIcaOiab+r8a0jabcMcaPiabc2faDnaaCaaaleqabaGaemOBa42aaSbaaWqaaiabicdaWaqabaaaaOGaeyypa0ZaamWaaeaadaWcaaqaaiabcIcaOiab+T7aSjabgUcaRiab+n7aNjabcMcaPiabdwgaLnaaCaaaleqabaGaeyOeI0IaeiikaGIae43UdWMaey4kaSIae43SdCMaeiykaKIae4hXdqhaaaGcbaGae43SdCMaey4kaSIae43UdWMaemyzau2aaWbaaSqabeaacqGHsislcqGGOaakcqGF7oaBcqGHRaWkcqGFZoWzcqGGPaqkcqGFepaDaaaaaaGccaGLBbGaayzxaaWaaWbaaSqabeaacqWGUbGBdaWgaaadbaGaeGimaadabeaaaaGccqGGUaGlcaWLjaGaaCzcamaabmaabaGaeGinaqJaeGyoaKdacaGLOaGaayzkaaaaaa@87E8@

Comparing the right-hand sides of the above two equations, one can show that provided *γ *<*λ*/*n*_0 _then Pr(monogeneric family) > Pr(monospecific genus) for *τ *less than some threshold value *τ*_0_, say; but for *τ *> *τ*_0 _the inequality is reversed. Thus as with the case of only background extinctions, monogeneric fossil families should be more common than monospecific fossil genera when the inter-cataclysm period is short. However if the inter-cataclysm period is longer the situation may be reversed.

## Concluding remarks

In the paper a number of analytic results on the size distributions of genera and families and on the probabilities of monospecific taxa have been derived under the assumption of a simple homogeneous birth-and-death model and various extinction scenarios. The results are incomplete due to the complexity of the analysis, especially in the case when both cataclysmic and background extinctions can occur. However it is hoped that there are sufficient results to enable testing of the birth-and-death model using empirical taxon size distributions obtained from the fossil record.

Undoubtedly more complex plausible extinction scenarios than the two extremes discussed in this paper could be considered. For example one could consider major extinction events which resulted in the destruction of a significant proportion (but not all) of species within a genus. However realistically formulating a model for this, not to mention its subsequent analysis, seems to present a formidable task.

One could also consider the size distribution of taxa existing over more than one inter-cataclysmic epoch. In this case one would need to consider mixtures of the distributions, using different (but assumed known) values of *τ*. In principle this is not difficult to do. If the durations of inter-cataclysmic epoch were not known one could consider *τ *as a random variable and consider the resulting infinite mixture. As a null model for catclysmic extinction events, it seems reasonable to assume that they occur independently at random, so that the time between two successive events would have an exponential distribution. An overall distribution for the size of a taxon could then be obtained by integrating the results obtained in the earlier sections with respect to an exponential density. This has been considered in another paper (Hughes and Reed[12]) where it is shown that, under certain conditions, the resulting size distributions exhibit fractal-like behaviour.

## Appendix

A point process {*X*_*t*_, *t *≥ 0} is said to be an *order statistic process *(Feigin[13]) if conditional on *X*_*τ *_- *X*_0 _= *k *the successive jump times (times of events) *T*_1_, *T*_2_,...,*T*_*k *_are distributed as the order statistics of *k *independent, identically distributed random variables with support on [0, *τ*]. The simplest example is when {*X*_*t*_} is a Poisson process, for which conditional on *X*_*τ *_- *X*_0 _= *k*, it is well known that the event times *T*_1_, *T*_2 _,..., *T*_*k *_have the same distribution as the order statistics of of *k *independent, uniformly distributed random variables on [0, *τ*].

For a given order statistic process the order statistic distribution can be shown (Feigin[13](Theorem 2)) to have cdf

F(t)=m(t)−m(0)m(τ)−m(0), 0≤y≤τ,     (50)
 MathType@MTEF@5@5@+=feaafiart1ev1aaatCvAUfKttLearuWrP9MDH5MBPbIqV92AaeXatLxBI9gBaebbnrfifHhDYfgasaacH8akY=wiFfYdH8Gipec8Eeeu0xXdbba9frFj0=OqFfea0dXdd9vqai=hGuQ8kuc9pgc9s8qqaq=dirpe0xb9q8qiLsFr0=vr0=vr0dc8meaabaqaciaacaGaaeqabaqabeGadaaakeaacqWGgbGrcqGGOaakcqWG0baDcqGGPaqkcqGH9aqpdaWcaaqaaiabd2gaTjabcIcaOiabdsha0jabcMcaPiabgkHiTiabd2gaTjabcIcaOiabicdaWiabcMcaPaqaaiabd2gaTjabcIcaOGGaciab=r8a0jabcMcaPiabgkHiTiabd2gaTjabcIcaOiabicdaWiabcMcaPaaacqGGSaalcqqGGaaicqaIWaamcqGHKjYOcqWG5bqEcqGHKjYOcqWFepaDcqGGSaalcaWLjaGaaCzcamaabmaabaGaeGynauJaeGimaadacaGLOaGaayzkaaaaaa@540E@

where *m*(*t*) = *E*(*X*_*t*_).

Puri[14] (Theorem 8) gives conditions for a non-homogeneous birth process, with birth rates *θ*_*i*_(*t*), to be an order statistic process. For the process {*K*_*t*_} (the number of genera) in second section, the birth rates *θ*_*k*_(*t*) are given by

*θ*_*k*_(*t*)*dt *= Pr (*K*(*t *+ *dt*) = *k *+ 1|*K*(*t*) = *k*)*dt *+ *o*(*dt*).     (51)

If we sum over *l *and *n *in (3) we find that with *p*_*k*_(*t*) = Pr{*K*_*t *_= *k*},

ddtpk(t)=γE(Lt|Kt=k−1)pk−1(t)−γE(Lt|Kt=k)pk(t)     (52)=θk−1(t)pk−1(t)−θk(t)pk(t)(53)
 MathType@MTEF@5@5@+=feaafiart1ev1aaatCvAUfKttLearuWrP9MDH5MBPbIqV92AaeXatLxBI9gBaebbnrfifHhDYfgasaacH8akY=wiFfYdH8Gipec8Eeeu0xXdbba9frFj0=OqFfea0dXdd9vqai=hGuQ8kuc9pgc9s8qqaq=dirpe0xb9q8qiLsFr0=vr0=vr0dc8meaabaqaciaacaGaaeqabaqabeGadaaakeaafaqaaeGaeaaaaeaadaWcaaqaaiabdsgaKbqaaiabdsgaKjabdsha0baacqWGWbaCdaWgaaWcbaGaem4AaSgabeaakiabcIcaOiabdsha0jabcMcaPaqaaiabg2da9aqaaGGaciab=n7aNjabdweafjabcIcaOiabdYeamnaaBaaaleaacqWG0baDaeqaaOGaeiiFaWNaem4saS0aaSbaaSqaaiabdsha0bqabaGccqGH9aqpcqWGRbWAcqGHsislcqaIXaqmcqGGPaqkcqWGWbaCdaWgaaWcbaGaem4AaSMaeyOeI0IaeGymaedabeaakiabcIcaOiabdsha0jabcMcaPiabgkHiTiab=n7aNjabdweafjabcIcaOiabdYeamnaaBaaaleaacqWG0baDaeqaaOGaeiiFaWNaem4saS0aaSbaaSqaaiabdsha0bqabaGccqGH9aqpcqWGRbWAcqGGPaqkcqWGWbaCdaWgaaWcbaGaem4AaSgabeaakiabcIcaOiabdsha0jabcMcaPiaaxMaacaWLjaaabaWaaeWaaeaacqaI1aqncqaIYaGmaiaawIcacaGLPaaaaeaaaeaacqGH9aqpaeaacqWF4oqCdaWgaaWcbaGaem4AaSMaeyOeI0IaeGymaedabeaakiabcIcaOiabdsha0jabcMcaPiabdchaWnaaBaaaleaacqWGRbWAcqGHsislcqaIXaqmaeqaaOGaeiikaGIaemiDaqNaeiykaKIaeyOeI0Iae8hUde3aaSbaaSqaaiabdUgaRbqabaGccqGGOaakcqWG0baDcqGGPaqkcqWGWbaCdaWgaaWcbaGaem4AaSgabeaakiabcIcaOiabdsha0jabcMcaPaqaamaabmaabaGaeGynauJaeG4mamdacaGLOaGaayzkaaaaaaaa@8BA1@

so that *K*_*t *_does evolve under a non-homogeneous birth process, with birth rates

*θ*_*k*_(*t*) = *γE*(*L*_*t*_|*K*_*t *_= *k*).     (54)

We now calculate *θ*_*k*_(*t*) explicitly. From Eq. (6),

pk(t)=(n0)k−1p(t)n0(k−1)![1−p(t)]k−1     (55)
 MathType@MTEF@5@5@+=feaafiart1ev1aaatCvAUfKttLearuWrP9MDH5MBPbIqV92AaeXatLxBI9gBaebbnrfifHhDYfgasaacH8akY=wiFfYdH8Gipec8Eeeu0xXdbba9frFj0=OqFfea0dXdd9vqai=hGuQ8kuc9pgc9s8qqaq=dirpe0xb9q8qiLsFr0=vr0=vr0dc8meaabaqaciaacaGaaeqabaqabeGadaaakeaacqWGWbaCdaWgaaWcbaGaem4AaSgabeaakiabcIcaOiabdsha0jabcMcaPiabg2da9maalaaabaGaeiikaGIaemOBa42aaSbaaSqaaiabicdaWaqabaGccqGGPaqkdaWgaaWcbaGaem4AaSMaeyOeI0IaeGymaedabeaakiabdchaWjabcIcaOiabdsha0jabcMcaPmaaCaaaleqabaGaemOBa42aaSbaaWqaaiabicdaWaqabaaaaaGcbaGaeiikaGIaem4AaSMaeyOeI0IaeGymaeJaeiykaKIaeiyiaecaaiabcUfaBjabigdaXiabgkHiTiabdchaWjabcIcaOiabdsha0jabcMcaPiabc2faDnaaCaaaleqabaGaem4AaSMaeyOeI0IaeGymaedaaOGaaCzcaiaaxMaadaqadaqaaiabiwda1iabiwda1aGaayjkaiaawMcaaaaa@59A4@

with *p*(*t*) = [(*λ *+ *γ*)*e*^-(*λ *+ *γ*)*t*^]/[*γ *+ *λe*^-(*λ *+ *γ*)*t*^] and we note for later use that

p′(t)p(t)=γ(λ+γ)γ+λe−(λ+γ)t.
 MathType@MTEF@5@5@+=feaafiart1ev1aaatCvAUfKttLearuWrP9MDH5MBPbIqV92AaeXatLxBI9gBaebbnrfifHhDYfgasaacH8akY=wiFfYdH8Gipec8Eeeu0xXdbba9frFj0=OqFfea0dXdd9vqai=hGuQ8kuc9pgc9s8qqaq=dirpe0xb9q8qiLsFr0=vr0=vr0dc8meaabaqaciaacaGaaeqabaqabeGadaaakeaadaWcaaqaaiqbdchaWzaafaGaeiikaGIaemiDaqNaeiykaKcabaGaemiCaaNaeiikaGIaemiDaqNaeiykaKcaaiabg2da9maalaaabaacciGae83SdCMaeiikaGIae83UdWMaey4kaSIae83SdCMaeiykaKcabaGae83SdCMaey4kaSIae83UdWMaemyzau2aaWbaaSqabeaacqGHsislcqGGOaakcqWF7oaBcqGHRaWkcqWFZoWzcqGGPaqkcqWG0baDaaaaaOGaeiOla4caaa@4D6D@

Since *p*_0_(*t*) = 0, we have

θ1(t)=−p′1(t)p1(t)=−n0p′(t)p(t)=γ(λ+γ)n0γ+λe−(λ+γ)t.     (56)
 MathType@MTEF@5@5@+=feaafiart1ev1aaatCvAUfKttLearuWrP9MDH5MBPbIqV92AaeXatLxBI9gBaebbnrfifHhDYfgasaacH8akY=wiFfYdH8Gipec8Eeeu0xXdbba9frFj0=OqFfea0dXdd9vqai=hGuQ8kuc9pgc9s8qqaq=dirpe0xb9q8qiLsFr0=vr0=vr0dc8meaabaqaciaacaGaaeqabaqabeGadaaakeaaiiGacqWF4oqCdaWgaaWcbaGaeGymaedabeaakiabcIcaOiabdsha0jabcMcaPiabg2da9iabgkHiTmaalaaabaGafmiCaaNbauaadaWgaaWcbaGaeGymaedabeaakiabcIcaOiabdsha0jabcMcaPaqaaiabdchaWnaaBaaaleaacqaIXaqmaeqaaOGaeiikaGIaemiDaqNaeiykaKcaaiabg2da9iabgkHiTmaalaaabaGaemOBa42aaSbaaSqaaiabicdaWaqabaGccuWGWbaCgaqbaiabcIcaOiabdsha0jabcMcaPaqaaiabdchaWjabcIcaOiabdsha0jabcMcaPaaacqGH9aqpdaWcaaqaaiab=n7aNjabcIcaOiab=T7aSjabgUcaRiab=n7aNjabcMcaPiabd6gaUnaaBaaaleaacqaIWaamaeqaaaGcbaGae83SdCMaey4kaSIae83UdWMaemyzau2aaWbaaSqabeaacqGHsislcqGGOaakcqWF7oaBcqGHRaWkcqWFZoWzcqGGPaqkcqWG0baDaaaaaOGaeiOla4IaaCzcaiaaxMaadaqadaqaaiabiwda1iabiAda2aGaayjkaiaawMcaaaaa@6C9E@

For *k *≥ 1 we have from (53) a difference equation to solve for *θ*_*k*_(*t*):

(*k *- 1)*θ*_*k *- 1_(*t*) - [1 - *p*(*t*)](*n*_0 _+ *k *- 2)*θ*_*k*_(*t*) = (*n*_0 _+ *k *-2){*n*_0 _[1 - *p*(*t*)] - (*k *- 1)*p*(*t*)}p′(t)p(t)
 MathType@MTEF@5@5@+=feaafiart1ev1aaatCvAUfKttLearuWrP9MDH5MBPbIqV92AaeXatLxBI9gBaebbnrfifHhDYfgasaacH8akY=wiFfYdH8Gipec8Eeeu0xXdbba9frFj0=OqFfea0dXdd9vqai=hGuQ8kuc9pgc9s8qqaq=dirpe0xb9q8qiLsFr0=vr0=vr0dc8meaabaqaciaacaGaaeqabaqabeGadaaakeaadaWcaaqaaiqbdchaWzaafaGaeiikaGIaemiDaqNaeiykaKcabaGaemiCaaNaeiikaGIaemiDaqNaeiykaKcaaaaa@35E0@.

By inspection, a solution of this equation is given by

*θ*_*k*_(*t*) = -p′(t)p(t)
 MathType@MTEF@5@5@+=feaafiart1ev1aaatCvAUfKttLearuWrP9MDH5MBPbIqV92AaeXatLxBI9gBaebbnrfifHhDYfgasaacH8akY=wiFfYdH8Gipec8Eeeu0xXdbba9frFj0=OqFfea0dXdd9vqai=hGuQ8kuc9pgc9s8qqaq=dirpe0xb9q8qiLsFr0=vr0=vr0dc8meaabaqaciaacaGaaeqabaqabeGadaaakeaadaWcaaqaaiqbdchaWzaafaGaeiikaGIaemiDaqNaeiykaKcabaGaemiCaaNaeiikaGIaemiDaqNaeiykaKcaaaaa@35E0@(*n*_0 _+ *k *- 1), *k *≥ 1.

As this solution gives the correct result (56) for *k *= 1 and a first-order linear difference equation needs only one boundary condition to uniquely determine the solution, we have proved that the birth rate is

θk(t)=γ(λ+γ)(n0+k−1)γ+λe−(λ+γ)t, k≥1.
 MathType@MTEF@5@5@+=feaafiart1ev1aaatCvAUfKttLearuWrP9MDH5MBPbIqV92AaeXatLxBI9gBaebbnrfifHhDYfgasaacH8akY=wiFfYdH8Gipec8Eeeu0xXdbba9frFj0=OqFfea0dXdd9vqai=hGuQ8kuc9pgc9s8qqaq=dirpe0xb9q8qiLsFr0=vr0=vr0dc8meaabaqaciaacaGaaeqabaqabeGadaaakeaaiiGacqWF4oqCdaWgaaWcbaGaem4AaSgabeaakiabcIcaOiabdsha0jabcMcaPiabg2da9maalaaabaGae83SdCMaeiikaGIae83UdWMaey4kaSIae83SdCMaeiykaKIaeiikaGIaemOBa42aaSbaaSqaaiabicdaWaqabaGccqGHRaWkcqWGRbWAcqGHsislcqaIXaqmcqGGPaqkaeaacqWFZoWzcqGHRaWkcqWF7oaBcqWGLbqzdaahaaWcbeqaaiabgkHiTiabcIcaOiab=T7aSjabgUcaRiab=n7aNjabcMcaPiabdsha0baaaaGccqGGSaalcqqGGaaicqWGRbWAcqGHLjYScqaIXaqmcqGGUaGlaaa@58B7@

Puri's [[14], Theorem 8] condition for an order-statistic process on (0, *τ*) requires the existence of a positive, continuous and integrable function, *h*(*t*) and positive constants *L*(*k*) for *k *= 1, 2,..., with *L*(1) = 1 such that

θk(t)exp⁡{∫0t[θk+1(u)−θk(u)]du}=h(t)L(k+1)L(k),
 MathType@MTEF@5@5@+=feaafiart1ev1aaatCvAUfKttLearuWrP9MDH5MBPbIqV92AaeXatLxBI9gBaebbnrfifHhDYfgasaacH8akY=wiFfYdH8Gipec8Eeeu0xXdbba9frFj0=OqFfea0dXdd9vqai=hGuQ8kuc9pgc9s8qqaq=dirpe0xb9q8qiLsFr0=vr0=vr0dc8meaabaqaciaacaGaaeqabaqabeGadaaakeaaiiGacqWF4oqCdaWgaaWcbaGaem4AaSgabeaakiabcIcaOiabdsha0jabcMcaPiGbcwgaLjabcIha4jabcchaWnaacmqabaWaa8qmaeaacqGGBbWwcqWF4oqCdaWgaaWcbaGaem4AaSMaey4kaSIaeGymaedabeaakiabcIcaOiabdwha1jabcMcaPiabgkHiTiab=H7aXnaaBaaaleaacqWGRbWAaeqaaOGaeiikaGIaemyDauNaeiykaKIaeiyxa0LaemizaqMaemyDauhaleaacqaIWaamaeaacqWG0baDa0Gaey4kIipaaOGaay5Eaiaaw2haaiabg2da9maalaaabaGaemiAaGMaeiikaGIaemiDaqNaeiykaKIaemitaWKaeiikaGIaem4AaSMaey4kaSIaeGymaeJaeiykaKcabaGaemitaWKaeiikaGIaem4AaSMaeiykaKcaaiabcYcaSaaa@639A@

In the present case this is satisfied with

*h*(*t*) = *n*_0_*γe*^(*λ*+*γ*)*t*^

and *L*(*k*) = (*n*_0 _- 1)_*k*_/n0k
 MathType@MTEF@5@5@+=feaafiart1ev1aaatCvAUfKttLearuWrP9MDH5MBPbIqV92AaeXatLxBI9gBaebbnrfifHhDYfgasaacH8akY=wiFfYdH8Gipec8Eeeu0xXdbba9frFj0=OqFfea0dXdd9vqai=hGuQ8kuc9pgc9s8qqaq=dirpe0xb9q8qiLsFr0=vr0=vr0dc8meaabaqaciaacaGaaeqabaqabeGadaaakeaacqWGUbGBdaqhaaWcbaGaeGimaadabaGaem4AaSgaaaaa@308B@. Also from Puri's Theorem 8,

E(Kt)=1+∫0th(u)du=1+n0γλ+γ[e(λ+γ)t−1].
 MathType@MTEF@5@5@+=feaafiart1ev1aaatCvAUfKttLearuWrP9MDH5MBPbIqV92AaeXatLxBI9gBaebbnrfifHhDYfgasaacH8akY=wiFfYdH8Gipec8Eeeu0xXdbba9frFj0=OqFfea0dXdd9vqai=hGuQ8kuc9pgc9s8qqaq=dirpe0xb9q8qiLsFr0=vr0=vr0dc8meaabaqaciaacaGaaeqabaqabeGadaaakeaacqWGfbqrcqGGOaakcqWGlbWsdaWgaaWcbaGaemiDaqhabeaakiabcMcaPiabg2da9iabigdaXiabgUcaRmaapedabaGaemiAaGMaeiikaGIaemyDauNaeiykaKIaemizaqMaemyDauhaleaacqaIWaamaeaacqWG0baDa0Gaey4kIipakiabg2da9iabigdaXiabgUcaRmaalaaabaGaemOBa42aaSbaaSqaaiabicdaWaqabaacciGccqWFZoWzaeaacqWF7oaBcqGHRaWkcqWFZoWzaaGaei4waSLaemyzau2aaWbaaSqabeaacqGGOaakcqWF7oaBcqGHRaWkcqWFZoWzcqGGPaqkcqWG0baDaaGccqGHsislcqaIXaqmcqGGDbqxcqGGUaGlaaa@5A56@

This agrees with the direct derivation of the expectation from the pgf of *K*_*t *_(10) and enables the computation of the joint distribution of the times of establishment of derived genera as that of the order statistics of a random sample of size *k *from a distribution with cdf

F(t)=e(λ+γ)t−1e(λ+γ)τ−1,0≤t≤τ.     (57)
 MathType@MTEF@5@5@+=feaafiart1ev1aaatCvAUfKttLearuWrP9MDH5MBPbIqV92AaeXatLxBI9gBaebbnrfifHhDYfgasaacH8akY=wiFfYdH8Gipec8Eeeu0xXdbba9frFj0=OqFfea0dXdd9vqai=hGuQ8kuc9pgc9s8qqaq=dirpe0xb9q8qiLsFr0=vr0=vr0dc8meaabaqaciaacaGaaeqabaqabeGadaaakeaafaqabeqacaaabaGaemOrayKaeiikaGIaemiDaqNaeiykaKIaeyypa0ZaaSaaaeaacqWGLbqzdaahaaWcbeqaaiabcIcaOGGaciab=T7aSjabgUcaRiab=n7aNjabcMcaPiabdsha0baakiabgkHiTiabigdaXaqaaiabdwgaLnaaCaaaleqabaGaeiikaGIae83UdWMaey4kaSIae83SdCMaeiykaKIae8hXdqhaaOGaeyOeI0IaeGymaedaaiabcYcaSaqaaiabicdaWiabgsMiJkabdsha0jabgsMiJkab=r8a0jabc6caUaaacaWLjaGaaCzcamaabmaabaGaeGynauJaeG4naCdacaGLOaGaayzkaaaaaa@55EA@

Thus it follows that at time *τ *the times since establishment of all derived genera are independent random variables with the truncated exponential distribution with pdf *f*_*K*_(*t*) given in (11).

To establish the truncated exponential nature of the distributions (*f*_*N*_(*t*) and *f*_*L*_(*t*), given in second section) of the times since establishment of species in respectively the pioneering genus and the pioneering family, is much easier. From the facts (established in the second section) that {*N*_*t*_} and {*L*_*t*_} are pure birth processes with both *N*_*t *_and *L*_*t *_having negative binomial distributions with *E*(*N*_*t*_) = *n*_0_*e*^*λt *^and E(*L*_*t*_) = *n*_0_*e*^(*λ*+*γ*)*t*^, and the well-known fact that a pure birth process is an order statistic process (Feigin[13]), one can easily establish (using (50)) the cdfs of the times since establishment of non-pioneering species in respectively the pioneeing genus and family. The pdfs, *f*_*N*_(*t*) and *f*_*L*_(*t*) given in (12) and (13) follow.

To establish the relationship (43) between the generating functions of *G*_*t *_(the number of genera which have existed by time *t*) and *L*_*t *_(the number of species which have existed by *t*), first let

*Y*_*t *_= *L*_*t *_- *n*_0 _and *Z*_*t *_= *G*_*t *_- 1     (58)

denote the numbers of derived species and genera respectively. Since any speciation could have given rise to a new genus with probability *p *= *γ*/(*λ*+*γ*), independently of other speciations, it follows that *Z*_*t*_|*y *~ Bin(*y*, *p*) and hence that

Pr(Gt=g|Lt=l)=Pr(Zt=g−1|Yt=l−n0)=(l−n0g−1)pg−1ql−n0−g+1=pg−1(g−1)!Dq(g−1)ql−n0     (59)
 MathType@MTEF@5@5@+=feaafiart1ev1aaatCvAUfKttLearuWrP9MDH5MBPbIqV92AaeXatLxBI9gBaebbnrfifHhDYfgasaacH8akY=wiFfYdH8Gipec8Eeeu0xXdbba9frFj0=OqFfea0dXdd9vqai=hGuQ8kuc9pgc9s8qqaq=dirpe0xb9q8qiLsFr0=vr0=vr0dc8meaabaqaciaacaGaaeqabaqabeGadaaakeaafaqaaeWadaaabaacbaGae8huaaLae8NCaiNaeiikaGIaem4raC0aaSbaaSqaaiabdsha0bqabaGccqGH9aqpcqWGNbWzcqGG8baFcqWGmbatdaWgaaWcbaGaemiDaqhabeaakiabg2da9iabdYgaSjabcMcaPaqaaiabg2da9aqaaiab=bfaqjab=jhaYjabcIcaOiabdQfaAnaaBaaaleaacqWG0baDaeqaaOGaeyypa0Jaem4zaCMaeyOeI0IaeGymaeJaeiiFaWNaemywaK1aaSbaaSqaaiabdsha0bqabaGccqGH9aqpcqWGSbaBcqGHsislcqWGUbGBdaWgaaWcbaGaeGimaadabeaakiabcMcaPaqaaaqaaiabg2da9aqaamaabmaabaqbaeqabiqaaaqaaiabdYgaSjabgkHiTiabd6gaUnaaBaaaleaacqaIWaamaeqaaaGcbaGaem4zaCMaeyOeI0IaeGymaedaaaGaayjkaiaawMcaaiabdchaWnaaCaaaleqabaGaem4zaCMaeyOeI0IaeGymaedaaOGaemyCae3aaWbaaSqabeaacqWGSbaBcqGHsislcqWGUbGBdaWgaaadbaGaeGimaadabeaaliabgkHiTiabdEgaNjabgUcaRiabigdaXaaaaOqaaaqaaiabg2da9aqaamaalaaabaGaemiCaa3aaWbaaSqabeaacqWGNbWzcqGHsislcqaIXaqmaaaakeaacqGGOaakcqWGNbWzcqGHsislcqaIXaqmcqGGPaqkcqGGHaqiaaGaemiraq0aa0baaSqaaiabdghaXbqaaiabcIcaOiabdEgaNjabgkHiTiabigdaXiabcMcaPaaakiabdghaXnaaCaaaleqabaGaemiBaWMaeyOeI0IaemOBa42aaSbaaWqaaiabicdaWaqabaaaaaaakiaaxMaacaWLjaWaaeWaaeaacqaI1aqncqaI5aqoaiaawIcacaGLPaaaaaa@8BEC@

where *q *= 1 - *p *and *D*_*q *_is the differential operator ddq
 MathType@MTEF@5@5@+=feaafiart1ev1aaatCvAUfKttLearuWrP9MDH5MBPbIqV92AaeXatLxBI9gBaebbnrfifHhDYfgasaacH8akY=wiFfYdH8Gipec8Eeeu0xXdbba9frFj0=OqFfea0dXdd9vqai=hGuQ8kuc9pgc9s8qqaq=dirpe0xb9q8qiLsFr0=vr0=vr0dc8meaabaqaciaacaGaaeqabaqabeGadaaakeaadaWcaaqaaiabdsgaKbqaaiabdsgaKjabdghaXbaaaaa@30C9@. Multiplying by the pmf *f*_*l *_= P(*L*_*t *_= *l*) and summing from *l *= *n*_0 _to ∞ yields the marginal pmf of *G*_*t*_, which can be written

Pr⁡(Gt=g)=pg−1(g−1)!Dq(g−1)q−n0∑l=n0∞qlfl=pg−1(g−1)!Dq(g−1)[Ψ˜(q)qn0]
 MathType@MTEF@5@5@+=feaafiart1ev1aaatCvAUfKttLearuWrP9MDH5MBPbIqV92AaeXatLxBI9gBaebbnrfifHhDYfgasaacH8akY=wiFfYdH8Gipec8Eeeu0xXdbba9frFj0=OqFfea0dXdd9vqai=hGuQ8kuc9pgc9s8qqaq=dirpe0xb9q8qiLsFr0=vr0=vr0dc8meaabaqaciaacaGaaeqabaqabeGadaaakeaafaqadeGadaaabaGagiiuaaLaeiOCaiNaeiikaGIaem4raC0aaSbaaSqaaiabdsha0bqabaGccqGH9aqpcqWGNbWzcqGGPaqkaeaacqGH9aqpaeaadaWcaaqaaiabdchaWnaaCaaaleqabaGaem4zaCMaeyOeI0IaeGymaedaaaGcbaGaeiikaGIaem4zaCMaeyOeI0IaeGymaeJaeiykaKIaeiyiaecaaiabdseaenaaDaaaleaacqWGXbqCaeaacqGGOaakcqWGNbWzcqGHsislcqaIXaqmcqGGPaqkaaGccqWGXbqCdaahaaWcbeqaaiabgkHiTiabd6gaUnaaBaaameaacqaIWaamaeqaaaaakmaaqahabaGaemyCae3aaWbaaSqabeaacqWGSbaBaaGccqWGMbGzdaWgaaWcbaGaemiBaWgabeaaaeaacqWGSbaBcqGH9aqpcqWGUbGBdaWgaaadbaGaeGimaadabeaaaSqaaiabg6HiLcqdcqGHris5aaGcbaaabaGaeyypa0dabaWaaSaaaeaacqWGWbaCdaahaaWcbeqaaiabdEgaNjabgkHiTiabigdaXaaaaOqaaiabcIcaOiabdEgaNjabgkHiTiabigdaXiabcMcaPiabcgcaHaaacqWGebardaqhaaWcbaGaemyCaehabaGaeiikaGIaem4zaCMaeyOeI0IaeGymaeJaeiykaKcaaOWaamWaaeaadaWcaaqaaiqbfI6azzaaiaGaeiikaGIaemyCaeNaeiykaKcabaGaemyCae3aaWbaaSqabeaacqWGUbGBdaWgaaadbaGaeGimaadabeaaaaaaaaGccaGLBbGaayzxaaaaaaaa@7B01@

where Ψ˜
 MathType@MTEF@5@5@+=feaafiart1ev1aaatCvAUfKttLearuWrP9MDH5MBPbIqV92AaeXatLxBI9gBaebbnrfifHhDYfgasaacH8akY=wiFfYdH8Gipec8Eeeu0xXdbba9frFj0=OqFfea0dXdd9vqai=hGuQ8kuc9pgc9s8qqaq=dirpe0xb9q8qiLsFr0=vr0=vr0dc8meaabaqaciaacaGaaeqabaqabeGadaaakeaacuqHOoqwgaacaaaa@2E4A@(·) is the pgf of *L*_*t *_which is the same as the pgf of *M*_*t *_(see (27)), but with *λ *replaced by *λ *+ *μ*. Thus

Pr⁡(Gt=g)=pg−1(g−1)!Dq(g−1)[Ψ˜(q;t)q]n0,     (60)
 MathType@MTEF@5@5@+=feaafiart1ev1aaatCvAUfKttLearuWrP9MDH5MBPbIqV92AaeXatLxBI9gBaebbnrfifHhDYfgasaacH8akY=wiFfYdH8Gipec8Eeeu0xXdbba9frFj0=OqFfea0dXdd9vqai=hGuQ8kuc9pgc9s8qqaq=dirpe0xb9q8qiLsFr0=vr0=vr0dc8meaabaqaciaacaGaaeqabaqabeGadaaakeaacyGGqbaucqGGYbGCcqGGOaakcqWGhbWrdaWgaaWcbaGaemiDaqhabeaakiabg2da9iabdEgaNjabcMcaPiabg2da9maalaaabaGaemiCaa3aaWbaaSqabeaacqWGNbWzcqGHsislcqaIXaqmaaaakeaacqGGOaakcqWGNbWzcqGHsislcqaIXaqmcqGGPaqkcqGGHaqiaaGaemiraq0aa0baaSqaaiabdghaXbqaaiabcIcaOiabdEgaNjabgkHiTiabigdaXiabcMcaPaaakmaadmaabaWaaSaaaeaacuqHOoqwgaacaiabcIcaOiabdghaXjabcUda7iabdsha0jabcMcaPaqaaiabdghaXbaaaiaawUfacaGLDbaadaahaaWcbeqaaiabd6gaUnaaBaaameaacqaIWaamaeqaaaaakiabcYcaSiaaxMaacaWLjaWaaeWaaeaacqaI2aGncqaIWaamaiaawIcacaGLPaaaaaa@5C25@

where (using (27))

Ψ˜(q;t)=β′(q−α′)+α′(β′−q)e−(λ+γ)(α′−β′)t(q−α′)+(β′−q)e−(λ+γ)(α′−β′)t.     (61)
 MathType@MTEF@5@5@+=feaafiart1ev1aaatCvAUfKttLearuWrP9MDH5MBPbIqV92AaeXatLxBI9gBaebbnrfifHhDYfgasaacH8akY=wiFfYdH8Gipec8Eeeu0xXdbba9frFj0=OqFfea0dXdd9vqai=hGuQ8kuc9pgc9s8qqaq=dirpe0xb9q8qiLsFr0=vr0=vr0dc8meaabaqaciaacaGaaeqabaqabeGadaaakeaacuqHOoqwgaacaiabcIcaOiabdghaXjabcUda7iabdsha0jabcMcaPiabg2da9maalaaabaacciGaf8NSdiMbauaacqGGOaakcqWGXbqCcqGHsislcuWFXoqygaqbaiabcMcaPiabgUcaRiqb=f7aHzaafaGaeiikaGIaf8NSdiMbauaacqGHsislcqWGXbqCcqGGPaqkcqWGLbqzdaahaaWcbeqaaiabgkHiTiabcIcaOiab=T7aSjabgUcaRiab=n7aNjabcMcaPiabcIcaOiqb=f7aHzaafaGaeyOeI0Iaf8NSdiMbauaacqGGPaqkcqWG0baDaaaakeaacqGGOaakcqWGXbqCcqGHsislcuWFXoqygaqbaiabcMcaPiabgUcaRiabcIcaOiqb=j7aIzaafaGaeyOeI0IaemyCaeNaeiykaKIaemyzau2aaWbaaSqabeaacqGHsislcqGGOaakcqWF7oaBcqGHRaWkcqWFZoWzcqGGPaqkcqGGOaakcuWFXoqygaqbaiabgkHiTiqb=j7aIzaafaGaeiykaKIaemiDaqhaaaaakiabc6caUiaaxMaacaWLjaWaaeWaaeaacqaI2aGncqaIXaqmaiaawIcacaGLPaaaaaa@75DD@

with *α' *and *β' *being the roots of (25) with *λ *replaced by *λ*+*μ*. The generating function of Ψ_*G*_(*s*; *t*) can be obtained by multiplying (60) above by *s*^*g *^and summing from *g *= 1 to ∞:

ΨG(s;t)=∑g=1∞sgPr⁡(Gt=g)=s∑g=1∞[sp]g−1(g−1)!dg−1dyg−1[Ψ˜(y;t)y]n0|y=q=s[Ψ˜(q+sp;t)q+sp]n0     (62)
 MathType@MTEF@5@5@+=feaafiart1ev1aaatCvAUfKttLearuWrP9MDH5MBPbIqV92AaeXatLxBI9gBaebbnrfifHhDYfgasaacH8akY=wiFfYdH8Gipec8Eeeu0xXdbba9frFj0=OqFfea0dXdd9vqai=hGuQ8kuc9pgc9s8qqaq=dirpe0xb9q8qiLsFr0=vr0=vr0dc8meaabaqaciaacaGaaeqabaqabeGadaaakeaafaqaaeWadaaabaGaeuiQdK1aaSbaaSqaaiabdEeahbqabaGccqGGOaakcqWGZbWCcqGG7aWocqWG0baDcqGGPaqkaeaacqGH9aqpaeaadaaeWbqaaiabdohaZnaaCaaaleqabaGaem4zaCgaaaqaaiabdEgaNjabg2da9iabigdaXaqaaiabg6HiLcqdcqGHris5aOGagiiuaaLaeiOCaiNaeiikaGIaem4raC0aaSbaaSqaaiabdsha0bqabaGccqGH9aqpcqWGNbWzcqGGPaqkaeaaaeaacqGH9aqpaeaadaabcaqaaiabdohaZnaaqahabaWaaSaaaeaacqGGBbWwcqWGZbWCcqWGWbaCcqGGDbqxdaahaaWcbeqaaiabdEgaNjabgkHiTiabigdaXaaaaOqaaiabcIcaOiabdEgaNjabgkHiTiabigdaXiabcMcaPiabcgcaHaaaaSqaaiabdEgaNjabg2da9iabigdaXaqaaiabg6HiLcqdcqGHris5aOWaaSaaaeaacqWGKbazdaahaaWcbeqaaiabdEgaNjabgkHiTiabigdaXaaaaOqaaiabdsgaKjabdMha5naaCaaaleqabaGaem4zaCMaeyOeI0IaeGymaedaaaaakmaadmaabaWaaSaaaeaacuqHOoqwgaacaiabcIcaOiabdMha5jabcUda7iabdsha0jabcMcaPaqaaiabdMha5baaaiaawUfacaGLDbaadaahaaWcbeqaaiabd6gaUnaaBaaameaacqaIWaamaeqaaaaaaOGaayjcSdWaaSbaaSqaaiabdMha5jabg2da9iabdghaXbqabaaakeaaaeaacqGH9aqpaeaacqWGZbWCdaWadaqaamaalaaabaGafuiQdKLbaGaacqGGOaakcqWGXbqCcqGHRaWkcqWGZbWCcqWGWbaCcqGG7aWocqWG0baDcqGGPaqkaeaacqWGXbqCcqGHRaWkcqWGZbWCcqWGWbaCaaaacaGLBbGaayzxaaWaaWbaaSqabeaacqWGUbGBdaWgaaadbaGaeGimaadabeaaaaaaaOGaaCzcaiaaxMaadaqadaqaaiabiAda2iabikdaYaGaayjkaiaawMcaaaaa@9BA4@

since the penultimate line is simply a Taylor series expansion about *q *of the last line.

Thus we conclude that

ΨG(s;t)=s[(λ+γ)λ+γsΨ˜(λ+γsλ+γ;t)]n0.     (63)
 MathType@MTEF@5@5@+=feaafiart1ev1aaatCvAUfKttLearuWrP9MDH5MBPbIqV92AaeXatLxBI9gBaebbnrfifHhDYfgasaacH8akY=wiFfYdH8Gipec8Eeeu0xXdbba9frFj0=OqFfea0dXdd9vqai=hGuQ8kuc9pgc9s8qqaq=dirpe0xb9q8qiLsFr0=vr0=vr0dc8meaabaqaciaacaGaaeqabaqabeGadaaakeaacqqHOoqwdaWgaaWcbaGaem4raCeabeaakiabcIcaOiabdohaZjabcUda7iabdsha0jabcMcaPiabg2da9iabdohaZnaadmaabaWaaSaaaeaacqGGOaakiiGacqWF7oaBcqGHRaWkcqWFZoWzcqGGPaqkaeaacqWF7oaBcqGHRaWkcqWFZoWzcqWGZbWCaaGafuiQdKLbaGaadaqadaqaamaalaaabaGae83UdWMaey4kaSIae83SdCMaem4CamhabaGae83UdWMaey4kaSIae83SdCgaaiabcUda7iabdsha0bGaayjkaiaawMcaaaGaay5waiaaw2faamaaCaaaleqabaGaemOBa42aaSbaaWqaaiabicdaWaqabaaaaOGaeiOla4IaaCzcaiaaxMaadaqadaqaaiabiAda2iabiodaZaGaayjkaiaawMcaaaaa@5CF3@
